# Supplementary material for: Systems immunology of transcriptional responses to viral infection identifies conserved antiviral pathways across macaques and humans
Source: Cell Rep. 2024 Jan 30;43(2):113706. doi: 10.1016/j.celrep.2024.113706 (PMC10915397; doi:10.1016/j.celrep.2024.113706)
Supplement: Document S1. Figures S1–S10 and Tables S4 and S5 [file mmc1.pdf]

Cell Reports, Volume *43*

Supplemental information

Systems immunology of transcriptional responses to viral infection identifies conserved antiviral pathways across macaques and humans

Kalani Ratnasiri, Hong Zheng, Jiaying Toh, Zhiyuan Yao, Veronica Duran, Michele Donato, Mario Roederer, Megha Kamath, John-Paul M. Todd, Matthew Gagne, Kathryn E. Foulds, Joseph R. Francica, Kizzmekia S. Corbett, Daniel C. Douek, Robert A. Seder, Shirit Einav, Catherine A. Blish, and Purvesh Khatri

SUPPLEMENTAL FIGURES and TABLES

SFig1: Gene pair correlation comparison across the macaques

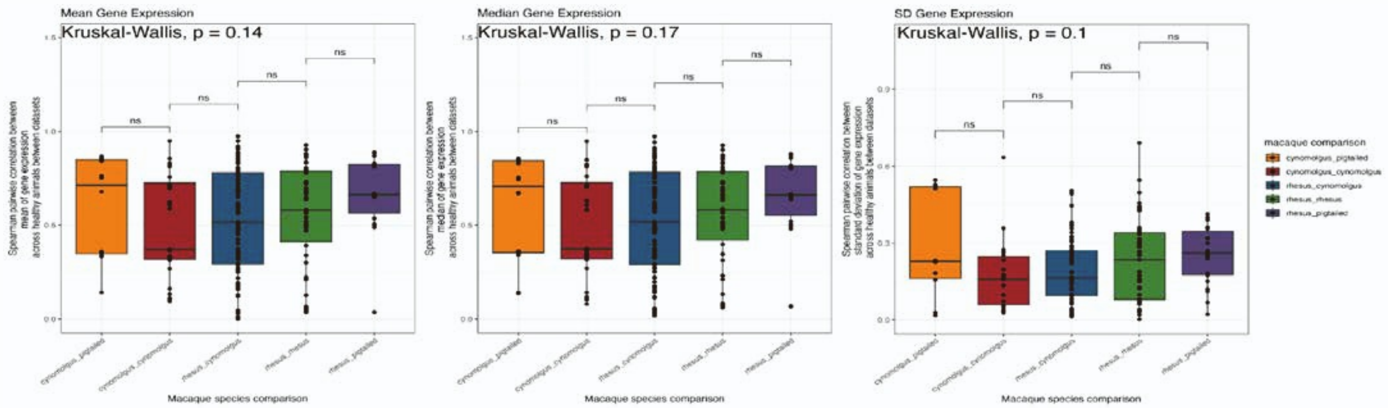

Correlation coefficients between the (left) mean (center) median and (right) standard deviation of gene expression of healthy animals per dataset in comparison to other datasets from the same or other macaque species. Pairwise wilcoxon test with bonferroni correction represented.

SFig2. AUROCs by dataset

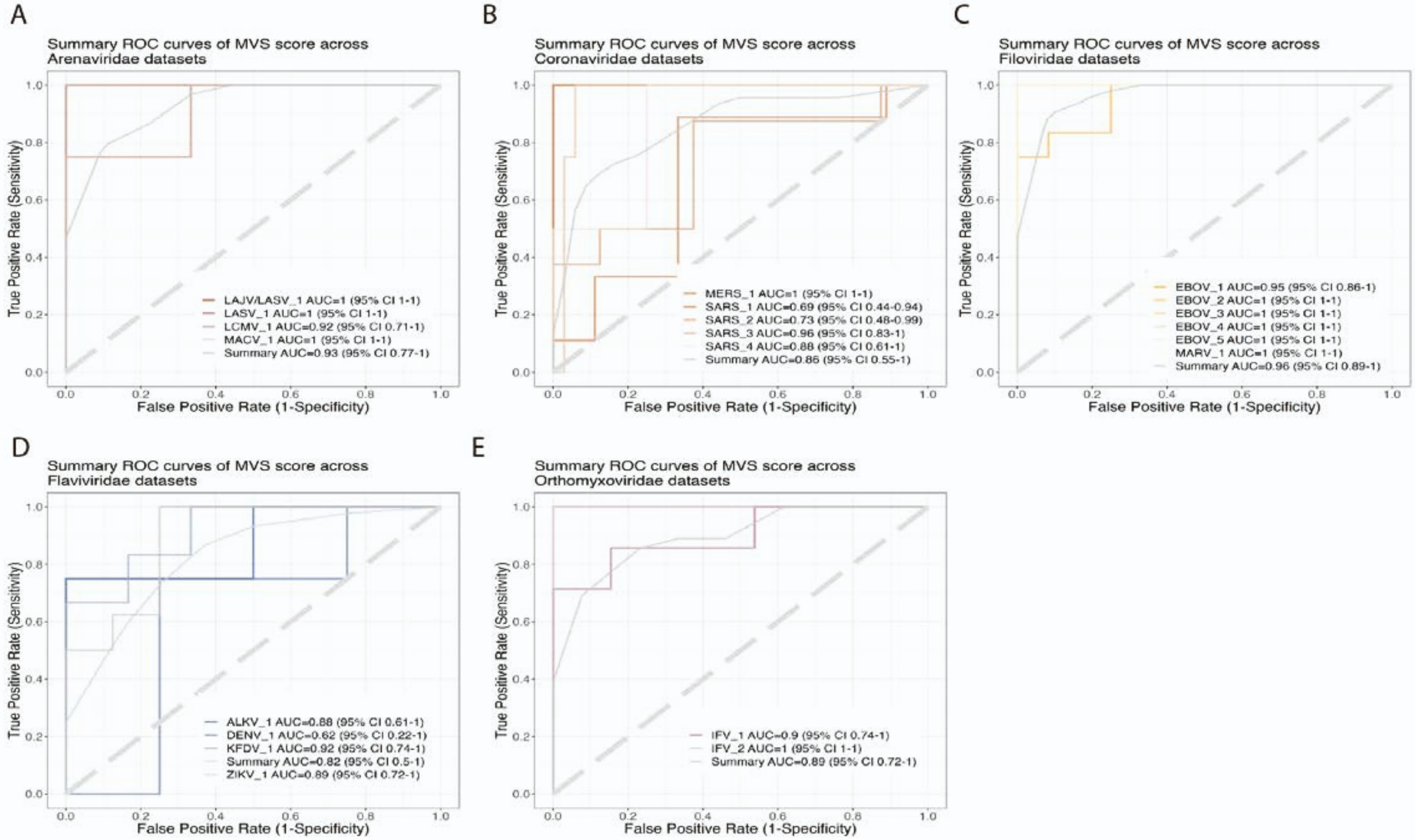

(A-E) ROC curves for distinguishing macaques with viral infection at peak MVS timepoint category from uninfected macaques, across datasets by viral family and colored by individual dataset.

SFig3. Validation of independent inflammation and monocyte and T cell function scores in NHP data

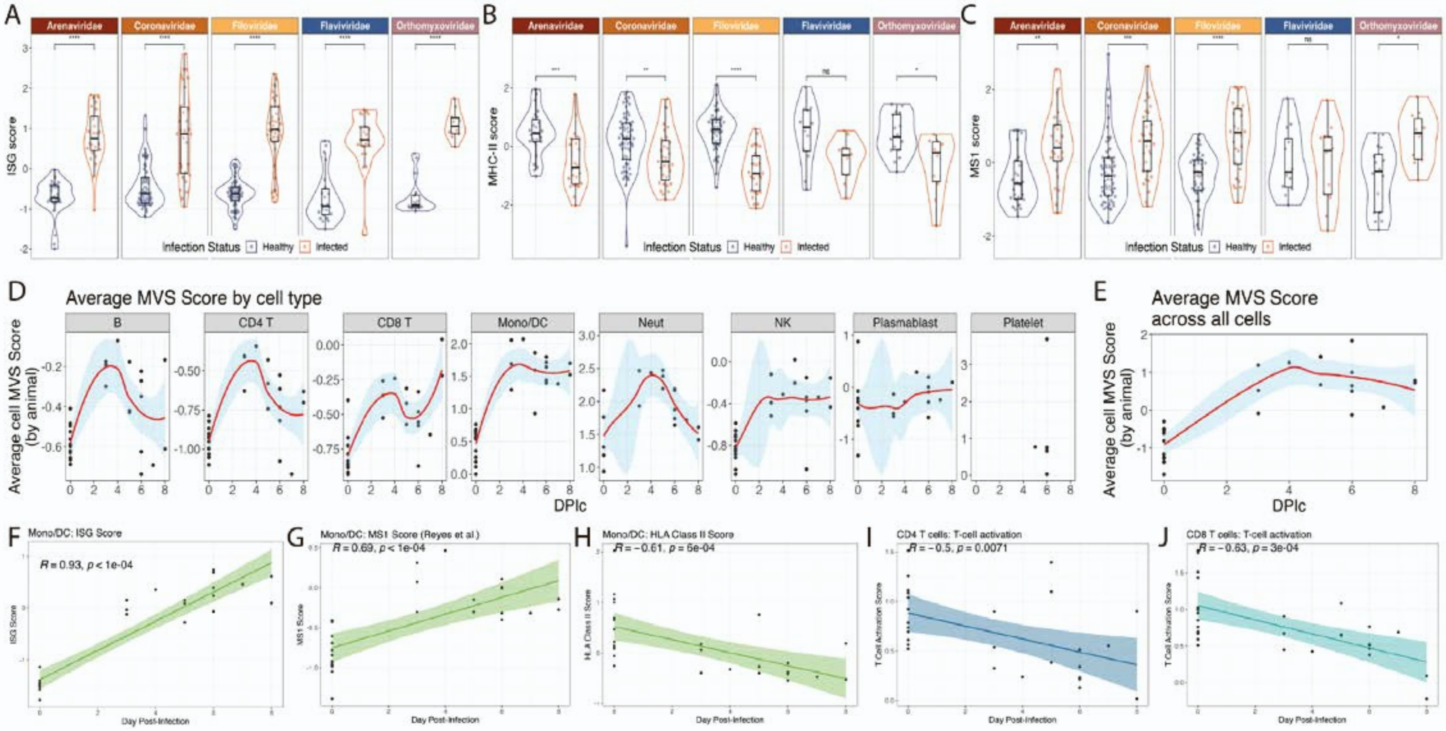

(A-J) Gene score by sample taking the geometric mean of the genes present and scaled across samples. **(A-C)** Distribution of the **(A)** ISG score, **(B)** HLA Class II Score and **(C)** MS1 Score comparing uninfected, healthy macaques to those at peak MVS score by viruses across five viral families. Each point represents a blood sample. Significance values were determined using an unpaired, one-sided Wilcoxon ranked-sum test with Bonferroni correction for multiple hypothesis testing. (**D–E)** Average MVS score by **(D)** cell type and **(E)** across all cells of the scRNA-seq EBOV infection NHP data from Figure 1H-K. **(F-J)** Correlation of average (**F)** ISG, **(G)** HLA class II and **(H)** MS1 scores in the Monocyte/DC population from Figure 1H with time post-infection. **(G-H)** Correlation of average T cell activation score in the **(I)** CD4 T cells and **(J)** CD8 T cell populations from Figure 1H with time post-infection.

SFig4. MVS score across all data timepoints

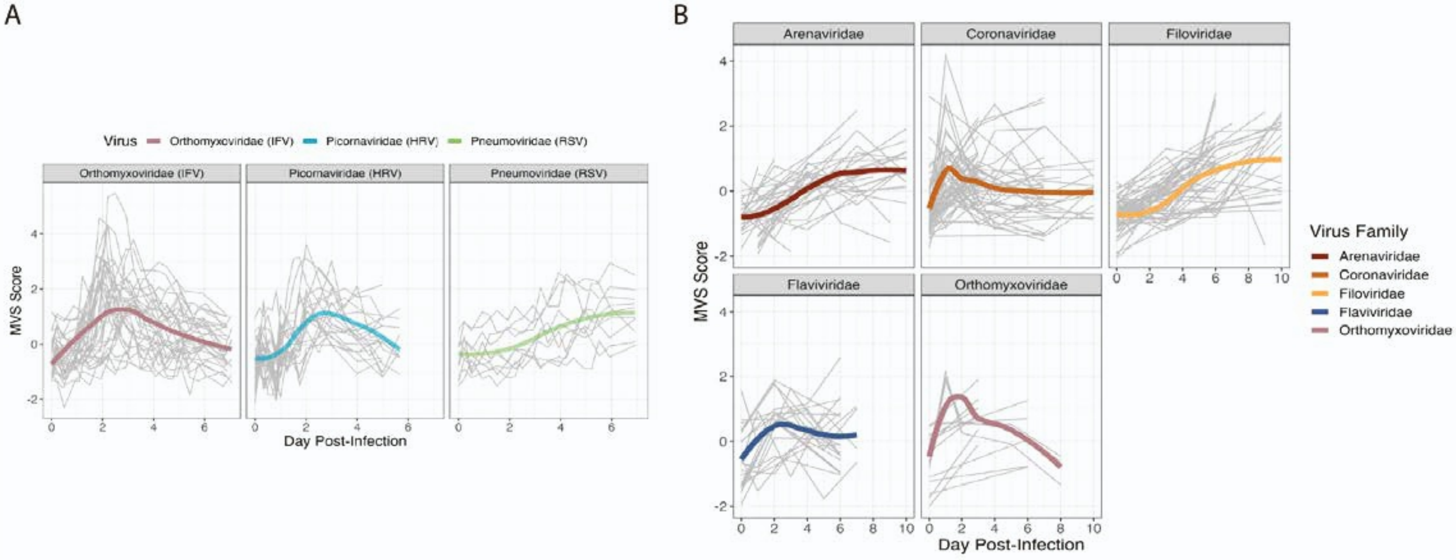

(A-B) MVS score calculated across (A) human longitudinal datasets and (B) all NHP longitudinal data.

Table S4: Time series analysis of MVS score by viral challenge in humans

Random effects:	Variance	Std. Deviation	Corr		
Participant	0.404	0.635			
Time	0.014	0.118	-0.36		
Residual	0.574	0.758			
**Fixed Effects:**	**β parameter estimate**	**Std. Error**	**t value**	**Pr(>|t|)**	
Intercept	-0.537	0.137	-3.932	1.70E-04	***
Time	0.904	0.059	15.443	< 2e-16	***
Time x Time	-0.133	0.008	-16.214	< 2e-16	***
Virus					
Picornaviridae (HRV)	-0.326	0.211	-1.547	0.126	
Pneumoviridae (RSV)	0.019	0.278	0.070	0.944	
Virus x Time					
Picornaviridae (HRV) x Time	0.145	0.112	1.288	0.198	
Pneumoviridae (RSV) x Time	-0.642	0.118	-5.465	6.45E-08	***
Virus x Time x Time					
Picornaviridae (HRV) x Time x Time	-0.032	0.019	-1.623	0.105	
Pneumoviridae (RSV) x Time x Time	0.134	0.016	8.234	5.43E-16	***
Number of observations:	1158				
Number of participants:	64				
AIC:	2918.891				

Mixed effects model using R package lmerTest. Comparison are to Orthomyxoviridae (IFV) challenge. Data from respiratory viral challenge of subjects with symptomatic disease. Timepoints included were from day 0 to day 7 post-virus challenge.
Signif. codes: 0 '***' 0.001 '**' 0.01 '*' 0.05

Table S5: Time series analysis of MVS score by Orthomyxoviridae challenge of humans and NHPs

**Random effects:**	**Variance**	**Std. Deviation**	**Corr**		
Subject	0.376	0.613			
Time	0.011	0.107	-0.24		
Residual	0.691	0.831			
**Fixed Effects:**	**β parameter estimate**	**Std. Error**	**t value**	**Pr(>|t|)**	
Intercept	-0.539	0.138	-3.908	2.56E-04	***
Time	0.905	0.063	14.397	< 2e-16	***
Time x Time	-0.133	0.009	-14.818	< 2e-16	***
Species					
NHP	0.153	0.289	0.530	0.597	
Species x Time					
NHP x Time	-0.003	0.250	-0.012	0.990385	
Species x Time x Time					
NHP x Time x Time	-0.010	0.045	-0.231	8.17E-01	
Number of observations:	656				
Number of participants:	48				
AIC:	1775.571				

Mixed effects model using R package lmerTest. NHP Orthomyxoviridae challenge cohorts compared to Human Orthomyxoviridae challenge cohorts. Timepoints included were from day 0 to day 7 post-virus challenge. Signif. codes: 0 '***' 0.001 '**' 0.01 '*' 0.05

SFig5. Meta-analysis of Human symptomatic challenge datasets by virus and time category

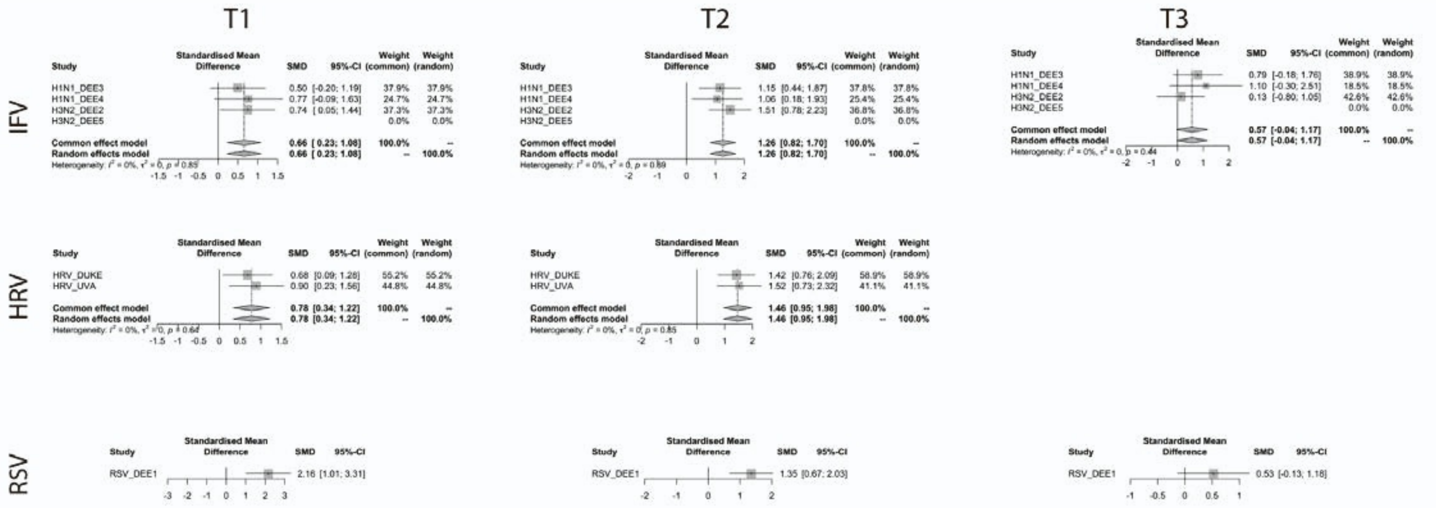

Meta-analysis of the MVS score across timepoints categories and viral infection in longitudinal human datasets.

SFig6. Meta-analysis of NHP datasets by virus and time category

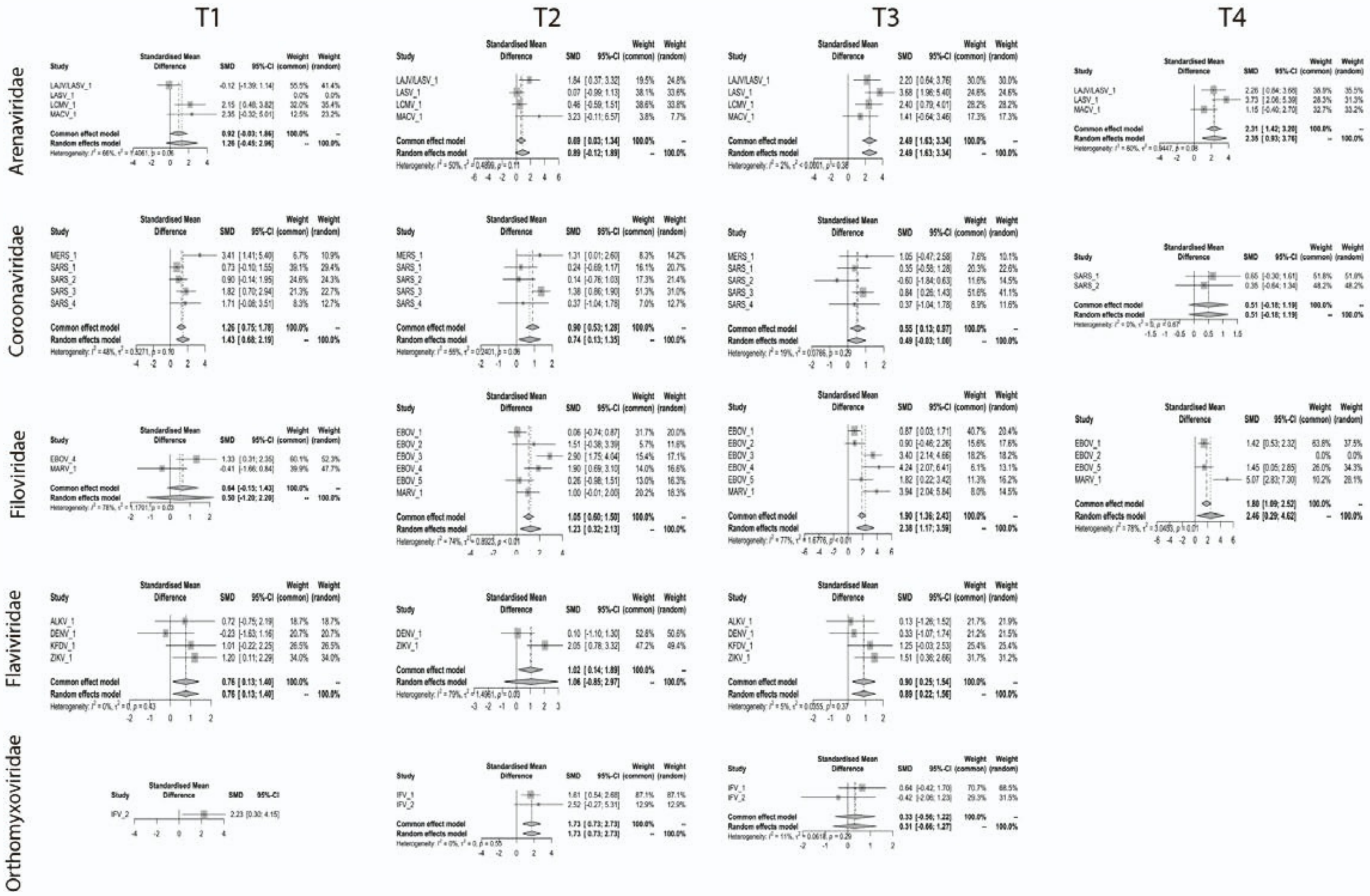

Meta-analysis of the MVS score across timepoints categories and viral infection in macaque datasets.

SFig7: BTM enrichment analysis across DEGs at peak timepoints per virus

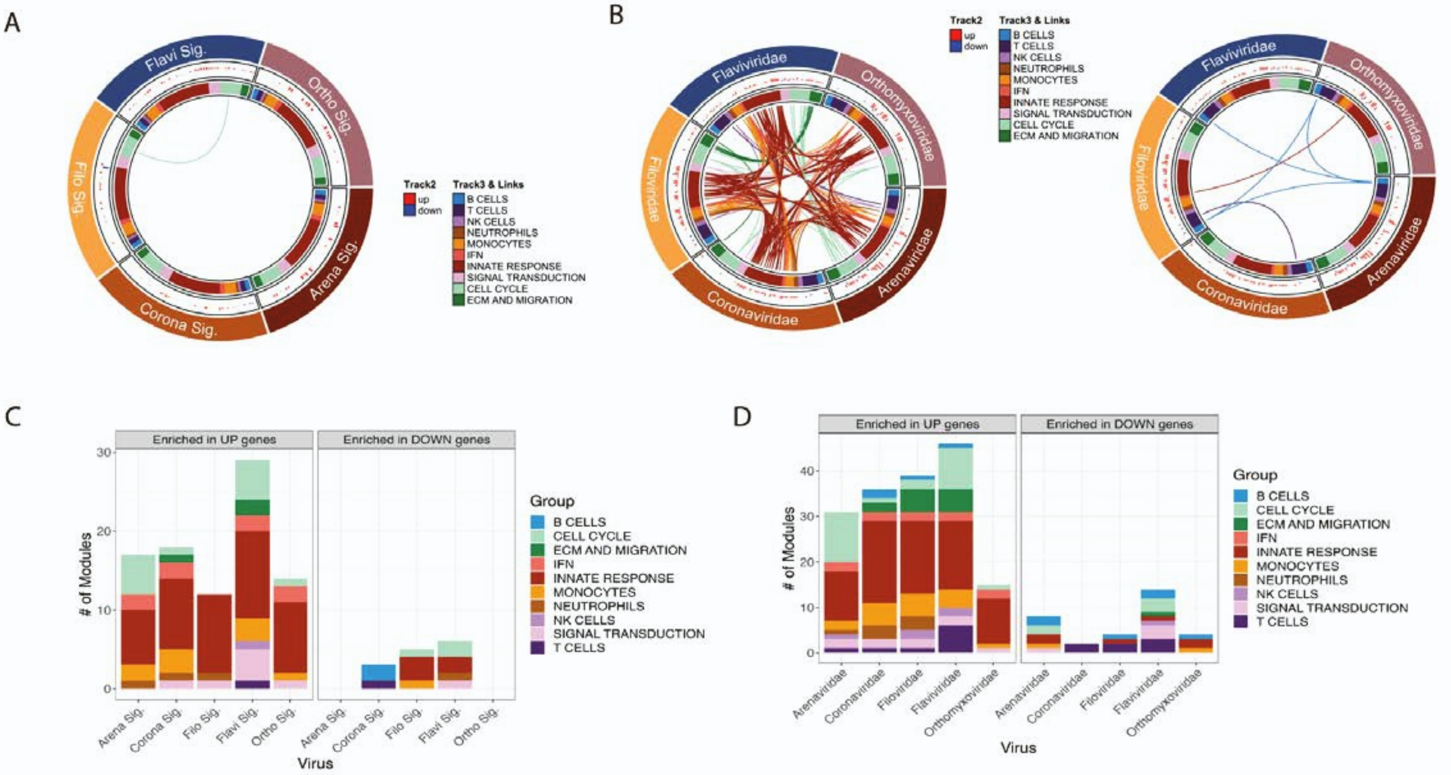

(A-C) Circos plots of BTM enrichment analysis across (A) underexpressed genes in each signature and **(B)** upregulated (left) and downregulated (right) genes at peak infection timepoints in NHP datasets. Each sector represents a viral family, each point in all the tracks represents a BTM that was significant in at least one virus (padj <0.1). Track 2 is a barplot of the geometric mean of the expression of the genes represented by the BTM and plotted where the BTM was significant (padj <0.1). Each color in Track 3 is a granular annotation for each BTM pathway. The inner track connects the same BTM across viral families if they are both (left) positively or (right) negatively enriched. **(C-D)** The count of the number of significant modules corresponding to each granular pathway by (C) overrepresented and underrepresented genes in each virus signature and (D) positive versus negative enriched genes by viral family - represented in the barplot.

SFig8. MVS and VRS in distinguishing between virus infected versus uninfected humans

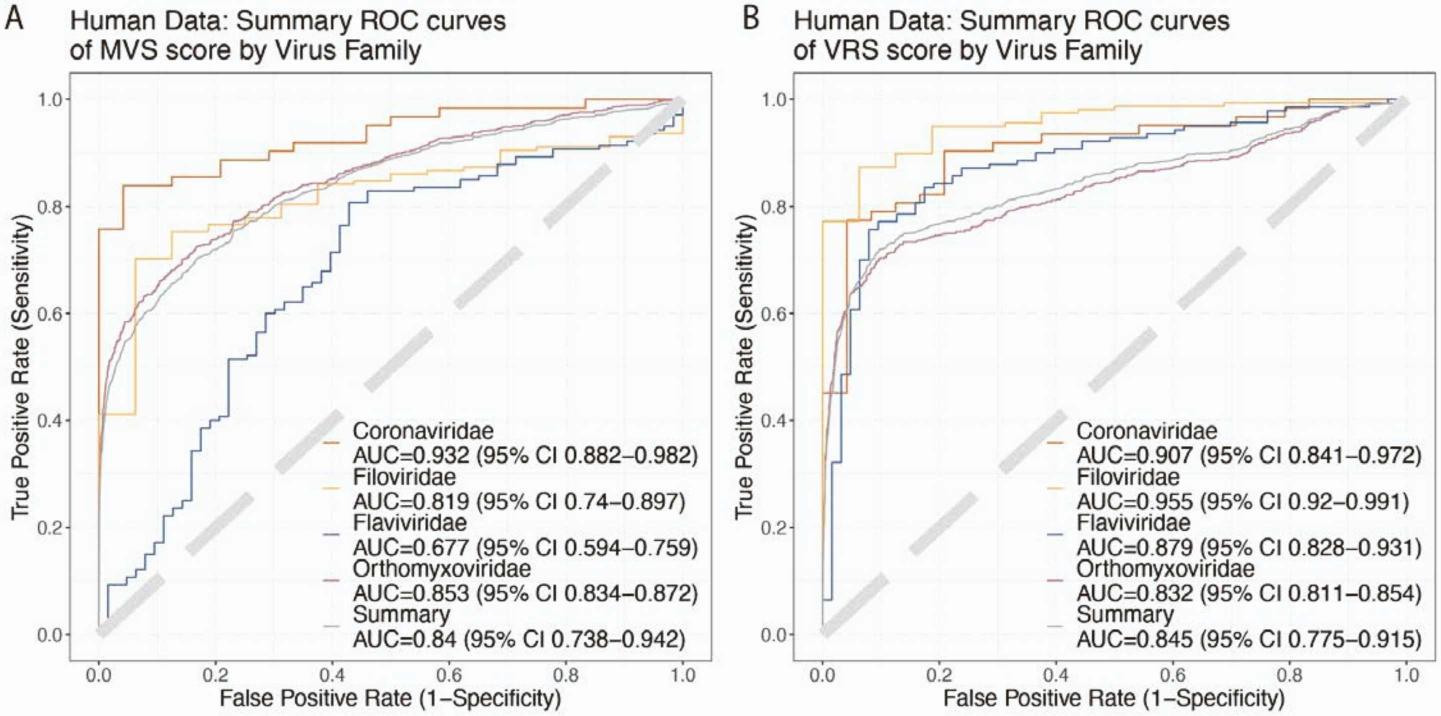

(A-B) ROC curves for distinguishing humans with viral infection from uninfected, healthy individuals split by viral family using the (A) MVS Score and (B) VRS Score.

SFig9. MVS score across human DNA and chronic viruses

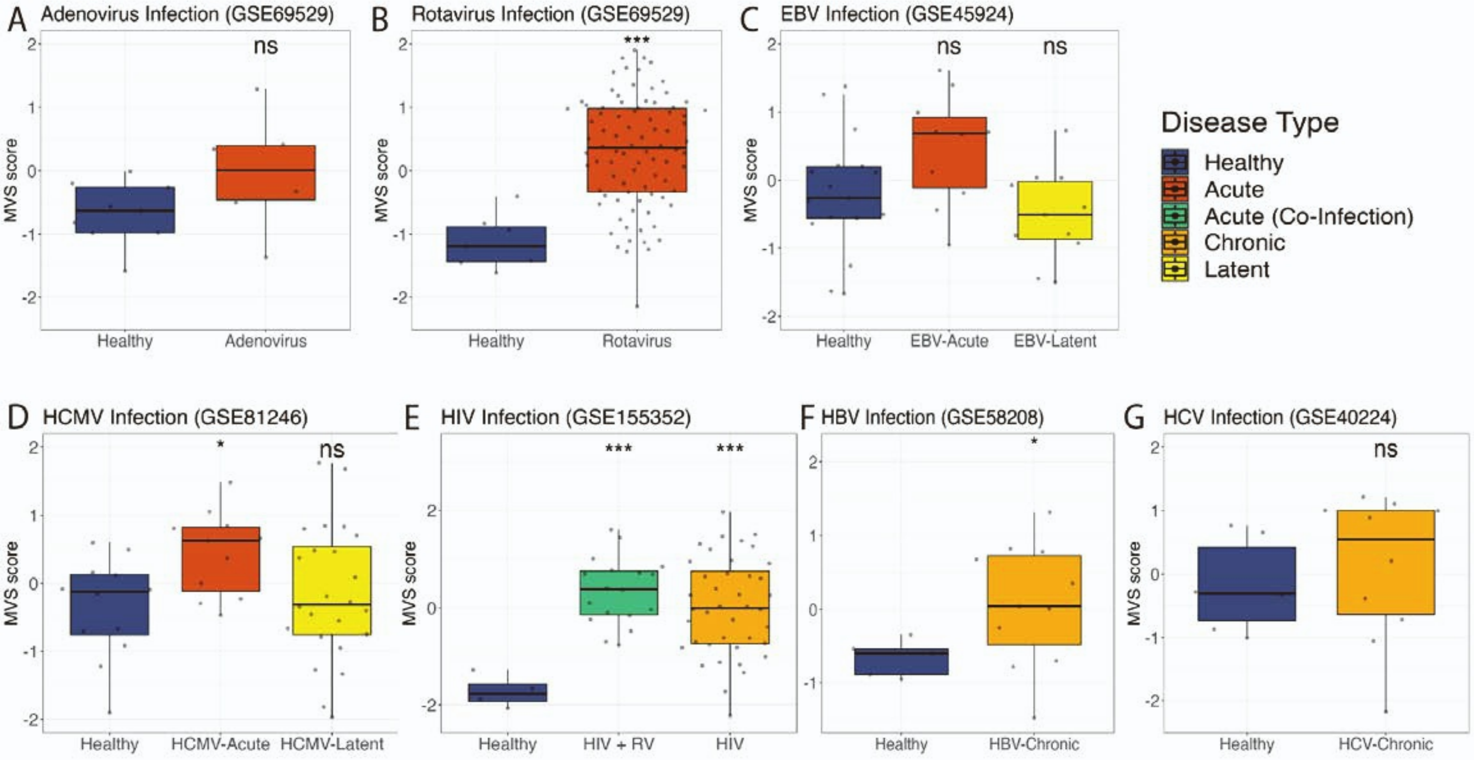

(A-G) MVS score in blood samples from healthy control subjects versus patients with **(A)** Adenovirus infection, **(B)** Rotavirus infection, **(C)** acute or latent EBV infection, **(D)** acute or latent HCMV infection, **(E)** HIV infection or HIV co-infection with a respiratory virus RV), **(F)** chronic HBV infection, and **(G)** chronic HCV infection. **(A-G)** Significance values were determined using an unpaired, one-sided Wilcoxon ranked-sum test comparing each condition to healthy samples.. Bonferroni correction for multiple hypothesis testing was applied per subfigure and significance values were assigned by asterisk. Asterisk values across figure are represented as follows: *p value < 0.05, **p value < 0.01, ***p value < 0.001, and ****p value < 0.0001. RV = respiratory virus.

SFig10: Overview of human scRNA-seq datasets

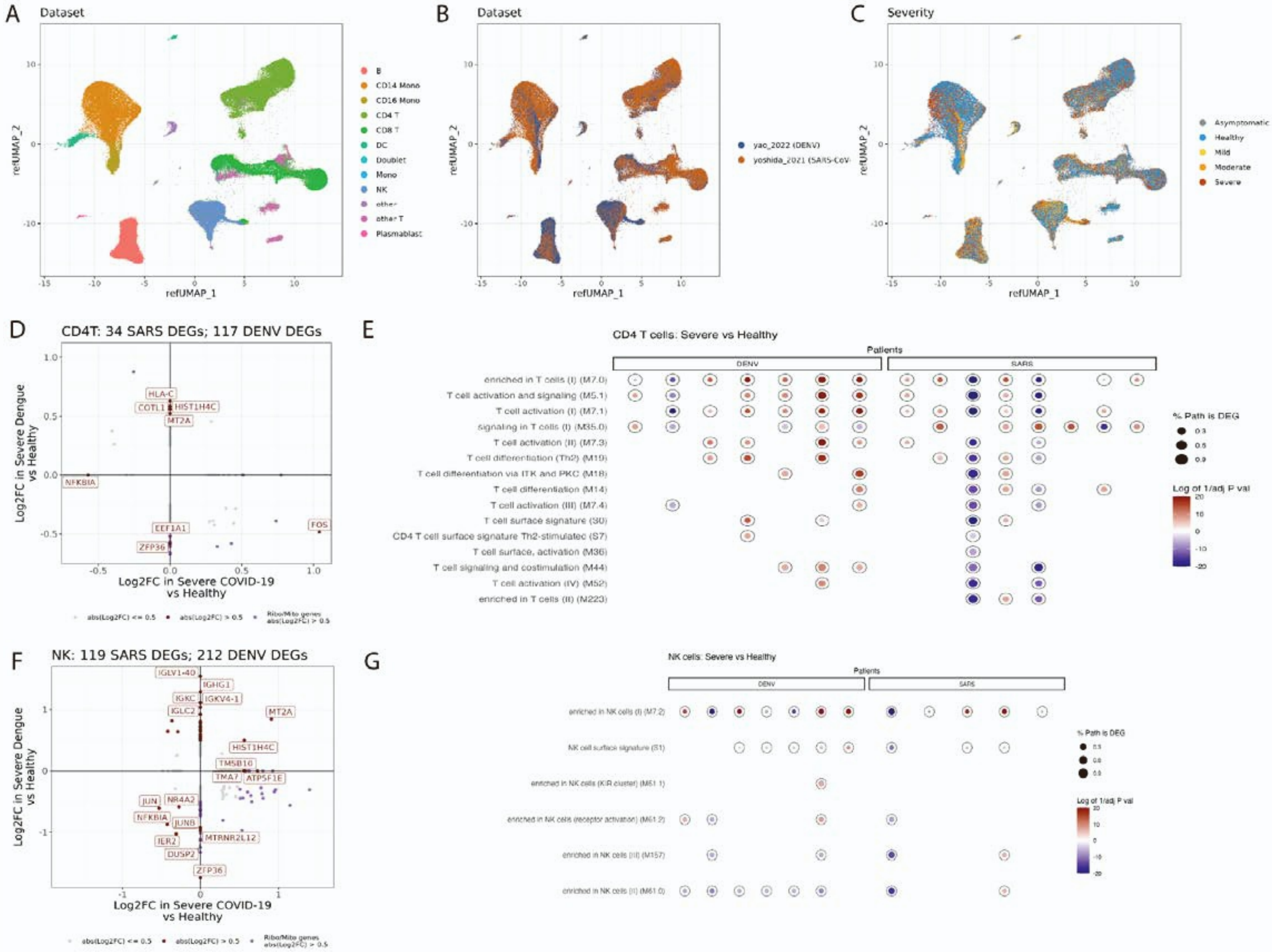

(A-C) UMAP visualization of immune cells from patients colored by **(A)** cell type, **(B)** dataset, and **(C)** patient severity. **(D and F)** Differential gene expression analysis of **(D)** CD4 T cells and **(F)** NK cells across scRNA-seq data from COVID-19 and dengue patients between patients with severe disease compared to healthy controls. **(E and G)** BTM enrichment analysis of differentially expressed genes from **(E)** CD4 T cells and **(G)** NK cells from each severe patient compared to the dataset's healthy patients.
