# Supplementary material for: Systems immunology of transcriptional responses to viral infection identifies conserved antiviral pathways across macaques and humans
Source: Cell Rep. 2024 Jan 30;43(2):113706. doi: 10.1016/j.celrep.2024.113706 (PMC10915397; doi:10.1016/j.celrep.2024.113706)
Supplement: Document S2. Article plus supplemental information [file mmc10.pdf]

Cell Reports

Article

Systems immunology of transcriptional responses to viral infection identifies conserved antiviral pathways across macaques and humans

Graphical abstract

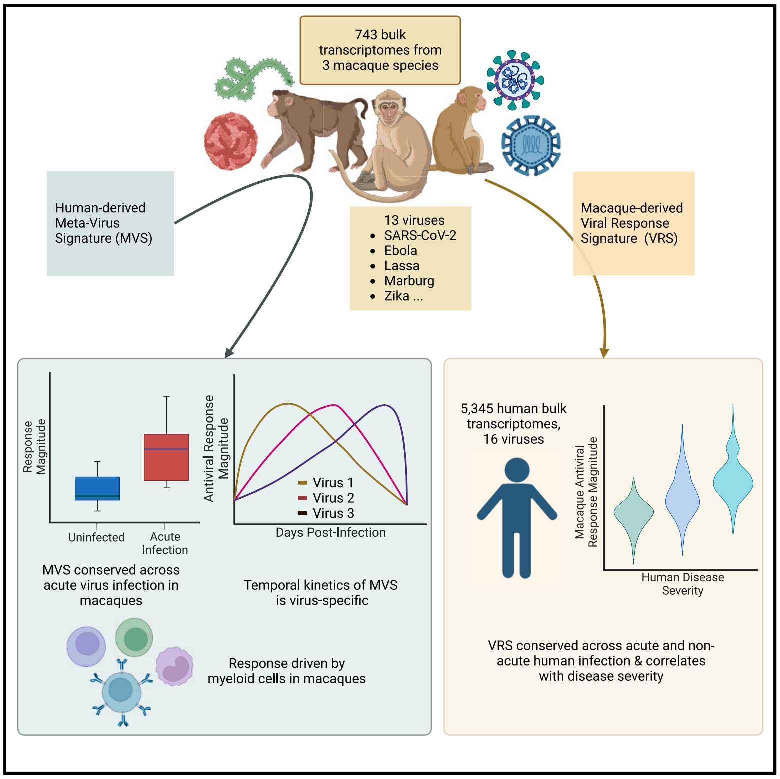

Highlights

- Virus-conserved response shared by humans and macaques
- Response dynamics are virus specific

Authors

Kalani Ratnasiri, Hong Zheng, Jiaying Toh, ..., Shirit Einav, Catherine A. Blish, Purvesh Khatri

Correspondence

pkhatri@stanford.edu

In brief

Transcriptomic data across viruses of pandemic potential do not exist in humans but do in macaque models. Ratnasiri et al. analyze >6,000 bulk and single-cell transcriptomic profiles from macaques and humans infected with one of 31 viruses to demonstrate antiviral host responses that are conserved across species and diverse viruses.

Ratnasiri et al., 2024, Cell Reports *43*, 113706
February 27, 2024 © 2024 The Authors.
https://doi.org/10.1016/j.celrep.2024.113706

CellPress

Cell Reports

CellPress
OPEN ACCESS

Article

Systems immunology of transcriptional responses to viral infection identifies conserved antiviral pathways across macaques and humans

Kalani Ratnasiri,[1,2] Hong Zheng,[4,5] Jiaying Toh,[1,3,4,5] Zhiyuan Yao,[6] Veronica Duran,[6] Michele Donato,[3,4] Mario Roederer,[7] Megha Kamath,[7] John-Paul M. Todd,[7] Matthew Gagne,[7] Kathryn E. Foulds,[7] Joseph R. Francica,[7] Kizzmekia S. Corbett,[7] Daniel C. Douek,[7] Robert A. Seder,[7] Shirit Einav,[6,8,9] Catherine A. Blish,[1,8,9,10,11] and Purvesh Khatri[3,4,5,11,12,*]

[1]Stanford Immunology Program, Stanford University School of Medicine, Stanford, CA 94305, USA
[2]Department of Epidemiology and Population Health, Stanford University, Stanford, CA 94305, USA
[3]Department of Surgery, Division of Abdominal Transplantation, Stanford University School of Medicine, Stanford, CA 94305, USA
[4]Center for Biomedical Informatics Research, Department of Medicine, Stanford University, Stanford, CA 94305, USA
[5]Institute for Immunity, Transplantation and Infection, Stanford University School of Medicine, Stanford, CA 94305, USA
[6]Department of Microbiology and Immunology, Stanford University, CA 94305, USA
[7]Vaccine Research Center, National Institute of Allergy and Infectious Diseases, National Institutes of Health, Bethesda, MD 20892, USA
[8]Department of Medicine, Stanford University School of Medicine, Stanford, CA 94305, USA
[9]Chan Zuckerberg Biohub, San Francisco, CA 94158, USA
[10]Medical Scientist Training Program, Stanford University School of Medicine, Stanford, CA 94305, USA
[11]Senior author
[12]Lead contact
*Correspondence: pkhatri@stanford.edu
https://doi.org/10.1016/j.celrep.2024.113706

SUMMARY

Viral pandemics and epidemics pose a significant global threat. While macaque models of viral disease are routinely used, it remains unclear how conserved antiviral responses are between macaques and humans. Therefore, we conducted a cross-species analysis of transcriptomic data from over 6,088 blood samples from macaques and humans infected with one of 31 viruses. Our findings demonstrate that irrespective of primate or viral species, there are conserved antiviral responses that are consistent across infection phase (acute, chronic, or latent) and viral genome type (DNA or RNA viruses). Leveraging longitudinal data from experimental challenges, we identify virus-specific response kinetics such as host responses to *Coronaviridae* and *Orthomyxoviridae* infections peaking 1–3 days earlier than responses to *Filoviridae* and *Arenaviridae* viral infections. Our results underscore macaque studies as a powerful tool for understanding viral pathogenesis and immune responses that translate to humans, with implications for viral therapeutic development and pandemic preparedness.

INTRODUCTION

Current, emerging, and reemerging viruses constantly threaten human health, not only by causing disease and death but also by driving wider societal and global consequences. Estimates suggest that RNA viruses make up to 44% of all emerging infectious diseases, with 2–3 novel virulent viruses discovered yearly and most of zoonotic origins.[1,2] Particular RNA viral families, including *Flaviviridae*, *Coronaviridae*, and *Orthomyxoviridae*, have led to multiple epidemics and pandemics within the 21st century,[3] demonstrating their pandemic potential. RNA viruses constantly evolve: mistake-prone RNA polymerases introduce genomic mutations, and zoonotic reservoirs drive unique evolutionary pressures on viruses that lead to unpredictable emergence patterns and disease manifestations.[4,5] While controlled human infection studies are ideal for developing translational solutions, such studies are generally difficult and unethical for lethal and emerging pathogens. Therefore, non-human primate (NHP) models, particularly the macaque model, continue to be critical for understanding disease pathogenesis, vaccine modalities, and therapeutic interventions.[6]

Previously, we identified a conserved host response in human infection across multiple viruses that have led to epidemics and pandemics.[7,8] However, multiple questions remain. For example, determining generalizability of host responses across infection by viruses such as Marburg and Lassa is needed; yet, the lack of available data on human infections caused by these and other viruses impedes the assessment of pan-viral responses. Additionally, understanding early antiviral responses remains important though complicated in-human profiling studies due to the challenge of identifying time of infection and virus incubation periods. Furthermore, while it is necessary to compare the longitudinal dynamics of host response induction across viruses, ethical concerns exist regarding human viral

© 2024 The Authors.
This is an open access article under the CC BY license (http://creativecommons.org/licenses/by/4.0/).

challenge studies. Here, macaque studies are advantageous because they allow for the understanding of diverse and lethal viral pathogens in well-controlled challenge studies, whereby measurements can be taken across multiple time points pre- and post-infection. However, the extent to which macaque immune responses reflect human host responses or vice versa is unclear, particularly whether both humans and macaques evoke similar antiviral responses upon infection. By leveraging transcriptomic profiles from both macaque and human infection studies, we aim to determine the utility of macaque models for understanding and predicting human responses to emerging viruses and to map conserved and unique features of the immune response to different viruses.

In this study, we performed the largest transcriptome analysis of viral disease in macaques and humans to date to (1) directly compare human and macaque antiviral responses and (2) to define host responses conserved across viruses of concern or specific to a virus. We used blood transcriptome data from 21 bulk RNA-seq datasets comprising 743 samples from 198 animals from three species of macaques (rhesus, cynomolgus, and pig-tailed macaques) and infection by 13 viruses across five viral families. We utilized longitudinal data to analyze the dynamics of viral response induction across numerous viruses, some of which have been seldom studied in the context of human transcriptomic responses. Further, we applied our previously identified conserved human host response across viruses, meta-virus signature (MVS) that distinguishes viral infection from healthy controls and predicts severity in humans, to show that macaques also induce antiviral responses similar to those of humans and that these response dynamics vary by viral family.[7,8] We also demonstrate that responses conserved across viruses in NHP data robustly translate to human transcriptomic responses to heterogeneous viral infections by leveraging 5,345 human samples across 47 datasets. Additionally, comparative analysis across antiviral responses identified differentiating features of T cell responses in *Flaviviridae* infection of macaques that were replicated in human studies. Together, this work demonstrates that macaque transcriptomic antiviral responses robustly recapitulate those in human viral disease and are conserved across diverse viruses, further supporting the use of macaque models to improve our understanding of host-virus interactions and underlying immune responses and to develop antiviral countermeasures, particularly in cases where human studies are not possible.

RESULTS

Data collection, curation, and preprocessing

We searched public repositories and publications for blood transcriptomic datasets from macaques with viral infection. We focused on acute RNA viruses from the World Health Organization (WHO) list of priority pathogens.[9] We also included *Orthomyxoviridae* due to its history of, and potential for, driving pandemics (Tables 1 and S1). We identified 21 datasets composed of 743 samples from 198 macaques infected with one of 13 viruses across five viral families (Tables 1 and S1). Together, these datasets represented a broad spectrum of biological and technical heterogeneity as they included data from three different macaque species infected with one of 13 viruses via different routes and doses and profiled using different microarray platforms and by RNA sequencing. We processed each dataset independently and utilized processed, normalized data when available. NHP datasets were not co-normalized. Because time points across datasets were not uniform, we grouped time points into six discrete categories, where T0 included uninfected samples prior to challenge, T1 spanned days 1–2 post infection, T2 was days 3–5, T3 was days 6–8, T4 was days 9–13, and T5 was days 14+ (Figure 1A). While most datasets included animals from baseline through infection, one *Arenaviridae* challenge dataset did not have pre-infection time points, requiring unpaired analyses when this dataset was included. Before analyzing all the macaque species together, we confirmed that the macaque species were comparable at baseline by comparing pairwise correlation of mean and median expression of the 3,055 shared genes across the different datasets. There were no differences in their expression correlation across datasets of different macaque species when compared to their correlation seen across datasets within the same macaque species (Figure S1).

Human antiviral response is conserved in macaques across diverse RNA virus infections

We first asked whether macaques are a representative model for studying human immune responses to viral infections. To answer this question, we used the MVS, the conserved immune response signature we have described and validated previously in acute human viral infections.[7,8] We chose the MVS instead of another set of genes such as interferon-stimulated genes (ISGs), because although ISGs have been repeatedly shown to be conserved across viral infections, they do not represent the full array of changes in the immune system in response to a virus. In contrast, we recently showed that the MVS includes differentially expressed genes from several immune cell types including innate (mature and immature neutrophils, monocytes, and dendritic cells) and adaptive (B, T, and natural killer [NK] cells) immune cells as well as ISGs.[8] In other words, the MVS enables for broad systemic comparisons of changes in the immune system.

As described previously,[7] we calculated the MVS score for each macaque sample in each dataset and compared the MVS scores at peak infection time points to those at the baseline. We defined peak infection time point in a dataset as the time point category with the highest median MVS score. The MVS scores were significantly higher (p adj < 0.001) and accurately distinguished macaques at peak infection from uninfected time points (area under the receiver operating characteristic [AUROC] curve ≥ 0.8) across all viruses (Figures 1B, 1C, and S2). We also examined other gene sets previously demonstrated to correlate with human viral infection. All viral infection datasets showed significant increase in ISG expression (p adj < 0.001), whereas *Arenaviridae*, *Coronaviridae*, *Filoviridae*, and *Orthomyxoviridae* infections demonstrated significant downregulation of major histocompatibility complex (MHC) class II genes (p adj < 0.05) and significant upregulation of MS1 signature genes (p adj < 0.01; Figures S3A, S3B, S3C, and Table S2).[10]

Importantly, we have shown that the MVS score is significantly correlated with severity of viral infection in humans.[8] There were

Table 1. Sample distribution of macaque bulk RNA-seq datasets

Variables	*Arenaviridae*	*Coronaviridae*	*Filoviridae*	*Flaviviridae*	*Orthomyxoviridae*	Totals
Total samples (% of all samples)	131 (17.6%)	267 (35.9%)	201 (27.1%)	98 (13.2%)	46 (6.2%)	743 (100%)
Total unique animals (% of all animals)	40 (20.2%)	61 (30.8%)	59 (29.8%)	22 (11.1%)	16 (8.1%)	198 (100%)
No. viral species	4 (LASV, LUJV, LCMV, MACV)	2 (MERS, SARS)	2 (EBOV, MARV)	4 (ALKV, DENV, KFDV, ZIKV)	1 (IFV)	13
No. datasets	4	5	6	4*	2	21
Variables (% of samples)	*Arenaviridae*	*Coronaviridae*	*Filoviridae*	*Flaviviridae*	*Orthomyxoviridae*	Totals (% of all samples)
Time point categories						
T0 (day 0)	26 (19.8%)	61 (22.8%)	59 (29.4%)	22 (22.4%)	16 (34.8%)	184 (24.8%)
T1 (days 1–2)	19 (14.5%)	40 (15%)	12 (6%)	22 (22.4%)	6 (13%)	99 (13.3%)
T2 (days 3–5)	26 (19.8%)	68 (25.5%)	46 (22.9%)	16 (16.3%)	10 (21.7%)	166 (22.3%)
T3 (days 6–8)	25 (19.1%)	39 (14.6%)	43 (21.4%)	22 (22.4%)	8 (17.4%)	137 (18.4%)
T4 (days 9–13)	32 (24.4%)	17 (6.4%)	23 (11.4%)	–	–	72 (9.7%)
T5 (days 14+)	3 (2.3%)	42 (15.7%)	18 (9%)	16 (16.3%)	6 (13%)	85 (11.4%)
Technology						
Microarray	113 (86.3%)	21 (7.9%)	157 (78.1%)	68 (69.4%)	46 (100%)	405 (54.5%)
RNA-seq	18 (13.7%)	246 (92.1%)	44 (21.9%)	30 (30.6%)	–	338 (45.5%)
Variables (% of animals)	*Arenaviridae*	*Coronaviridae*	*Filoviridae*	*Flaviviridae*	*Orthomyxoviridae*	Totals (% of all samples)
Macaque species						
Cynomolgus (*Macaca fascicularis*)	29 (72.5%)	–	42 (71.2%)	4 (18.2%)	–	75 (37.9%)
Pig-tailed (*Macaca nemestrina*)	–	–	–	8 (36.4%)	–	8 (4%)
Rhesus (*Macaca mulatta*)	11 (27.5%)	61 (100%)	17 (28.8%)	10 (45.5%)	16 (100%)	115 (58.1%)

The asterisk denotes dataset GSE185797 that was split into two, GSE185797_KFDV and GSE185797_ALKV, because it included two independent virus infection model-associated baseline (day 0) controls.

four datasets with known risk factors for severity in macaques. The MVS score was significantly associated with known risk factors ($p < 0.04$), including Machupo virus infection of unvaccinated (more severe disease) versus vaccinated macaques[11] (Figure 1D), infection by Mayinga (more severe disease) versus Makona Ebola strains[12,13] (Figure 1E), influenza infection of old (more severe disease) versus young macaques[14] (Figure 1F), and infection by live (more severe disease) versus inactivated influenza infection[15] (Figure 1G). These results demonstrate that conserved transcriptional signatures to viral infections in humans are also conserved in macaques and associated with known risk factors for severe outcome.

Next, we sought to investigate whether the conserved antiviral response in macaques is driven by the same immune cells as reported in humans. We previously found that the MVS response is primarily driven by myeloid cells in humans with COVID-19.[8] Therefore, we utilized the only available public NHP dataset of single-cell RNA sequencing (scRNA-seq) of blood cells in acute viral infection (GSE158390) to see if similar cell types were responsible for MVS responses in NHPs. This scRNA-seq dataset of whole blood samples from macaques infected with Ebola virus (56,929 cells from 17 macaques)[16] included pre-infection time points and multiple days post infection (DPI) for which we calculated an average MVS score per cell (Figures 1H, 1I, and 1J). Taking an average of MVS scores per cell type and time point, we found that, similar to humans with COVID-19, the MVS genes were preferentially expressed in myeloid cells across all timepoints[8] (Figure 1K). While the expression of MVS greatly varies by cell type, it increases in most cell types after infection (Figures S3D and S3E).

To further characterize infection-driven changes in myeloid cells, we examined longitudinal gene expression profiles from pre-infection to 8 DPI when macaques developed severe and/or fatal disease. We observed an increase in ISG expression ($r = 0.93$, $p < 1e{-}4$; Figure S3F) and MS1 signature genes ($r = 0.69$, $p < 1e{-}4$; Figure S3G) and a decrease in MHC class II genes ($r = -0.61$, $p < 6e{-}4$; Figure S3H). These changes have also been observed in myeloid cells in patients with severe COVID-19 and influenza.[10,17,18] Additionally, upregulation of MS1 genes and downregulation of MHC class II is consistent with the acquisition of a myeloid-derived suppressor cell (MDSC)-like phenotype, which in turn suppresses T cell activation. Therefore, we evaluated changes in T cell activation across infection.[19] We observed significant downregulation of T cell activation genes in both CD4 and CD8 T cell subsets ($r < -0.5$, $p < 0.008$; Figures S3I and S3J) from pre-infection to day 8

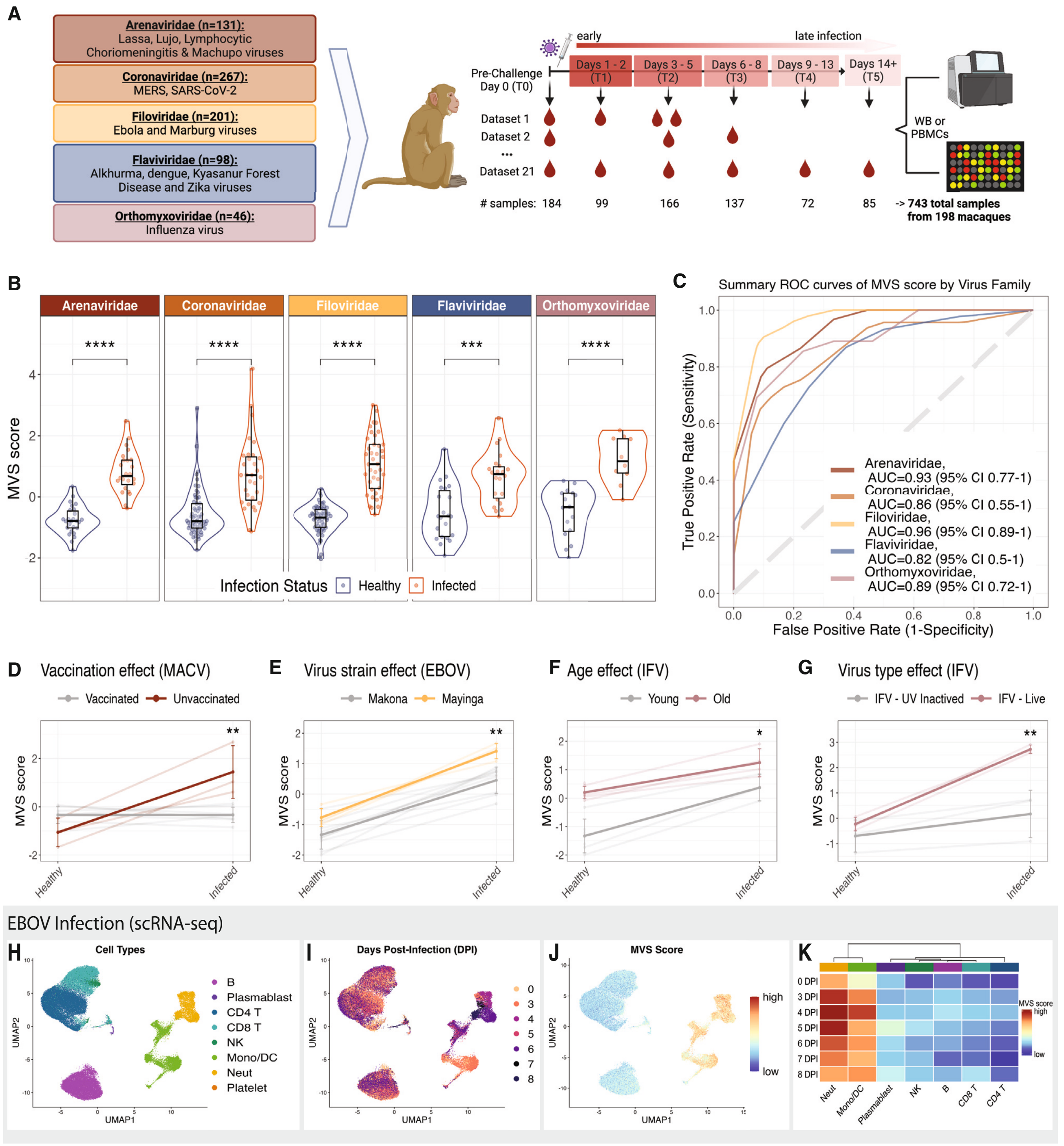

Figure 1. Human antiviral host response is conserved during viral infection in macaques and driven by myeloid cells

(A) Schematic of macaque sample overview and time point distribution.

(B) Distribution of the meta-virus signature (MVS) scores comparing uninfected, healthy macaques to those at peak MVS score by viruses across five viral families. Each point represents a blood sample. Data are displayed as both violin plot and box and whisker plots. The whisker above the box plot extends from upper quartile to the highest value within the 75th percentile + 1.5*interquartile range. The whisker below the box extends from the lower quartile the the lowest actual value that is within the 25th percentile + 1.5*interquartile range. Significance values were determined using an unpaired, one-sided Wilcoxon ranked-sum test with Bonferroni correction for multiple hypothesis testing.

(C) ROC curves for distinguishing macaques with viral infection at peak MVS time point category from uninfected macaques, colored by the viral family associated with infection (382 samples in 21 datasets).

(legend continued on next page)

post infection. Together, these results reveal that the dynamics described in severe human viral diseases are also present in critical/fatal Ebola infection of rhesus macaques, further supporting our hypothesis that immune cell responses are consistent across viral and host species in RNA virus infections.

Together, these data provide strong evidence that the conserved immune response to viral infections in humans is also conserved in macaques and primarily driven by myeloid cells. Similar to humans, our analysis further suggests that it may be correlated with severity of infection in macaques.

Temporal patterns of the conserved antiviral responses differ by viral families in humans and macaques

Because the peak infection time point differed for each virus, we investigated whether temporal patterns of the host response differed by virus in humans and macaques. First, we identified seven human challenge studies (GSE73072) where participants were inoculated with either influenza (IFV; family: *Orthomyxoviridae*), human rhinovirus (HRV; family: *Picornaviridae*), or respiratory syncytial virus (RSV; family: *Pneumoviridae*), and transcriptional data were collected from blood samples pre- and post-infection (Table S3). We excluded participants that were asymptomatic and did not shed virus (i.e., uninfected). We calculated the MVS score at all time points collected in symptomatic infected patients (Figures 2A and S4A) and assessed temporal changes in the MVS score with different viral infections (Figure 2B and Table S4). While IFV and HRV infections had highest MVS scores between days 1–5 post infection, RSV infection showed peak MVS scores at later time points, between days 3–7 (Figures 2A, 2B, and S5). A mixed-effects model with time as a continuous variable also showed that dynamics of the MVS in RSV-infected patients differed significantly ($p < 0.001$) from those of patients with IFV or HRV infections (Table S4).

Next, we investigated whether similar virus-dependent differences in the kinetics of the MVS were also present in macaques (Figures 2C, S4B, and S6). Similar to IFV infection in humans, *Orthomyxoviridae* infection of macaques had early peak MVS responses at 1–3 DPI (Figures 2C and 2D). We further comparatively assessed response dynamics via mixed-effects modeling using macaque infection by *Orthomyxoviridae* viruses as the comparator; however, we limited our analysis to pre-infection to day 7 post infection as *Flaviviridae* datasets had no time points past day 7 (Table 2). Temporal MVS responses in macaque infection by *Arenaviridae* and *Filoviridae* viruses were significantly different from those by *Orthomyxoviridae* infection ($p < 0.01$), whereas infection by *Coronaviridae* and *Flaviviridae* viruses was less significant but did differ from *Orthomyxoviridae* infection dynamics ($p < 0.05$; Table 2). For example, while *Orthomyxoviridae* and *Coronaviridae* only showed significant differences in MVS Scores at T1 and T2 compared to baseline ($p < 0.05$), *Filoviridae* and *Arenaviridae* infections showed the most significant differences in MVS score compared to baseline at T3 ($p < 0.001$; Figure 2D). While we tried mixed-effects models that included sample type (whole blood or peripheral blood mononuclear cells [PBMCs]), sequencing method (microarray or RNA-seq), and macaque species, we found that the simpler model that included virus type and DPI better explained the data. There was no significant effect of sample type on MVS magnitude, which is not unexpected: while PBMC preps deplete neutrophils, we have previously shown that $CD14^+$ monocytes increase in proportion and drive the MVS signal; hence, the MVS signature goes up with and without neutrophils present.

We also compared the temporal MVS response between macaques and humans infected with *Orthomyxoviridae* for which there were data across both species and found that infection time point, not species, drove response magnitude (Table S5), further suggesting conserved antiviral response dynamics across species. Overall, these data highlight that MVS is robustly conserved in humans and macaques across viruses, though host response dynamics differ by virus type, which may be important for understanding viral incubation and latency periods.

Unbiased transcriptomic analysis of NHP demonstrates conserved antiviral responses to acute RNA viruses that translate to humans

Transcriptomic data for certain viral diseases (e.g., Lassa virus, Machupo virus, Kyasanur forest disease virus) in humans are not publicly available, nor are data collected at early stages of many lethal and newly emerging viral diseases. In such cases, macaque studies provide the only immediate transcriptomic data to learn primate immune responses. Therefore, we investigated whether macaque antiviral responses were similarly conserved across viral infection and translatable to human viral infections (Figure 3A). First, we determined differentially expressed genes (DEGs) across datasets per viral family by time point category compared to the baseline T0 to identify peak infection time points in an unbiased way (Figure 3B and Table S6). From here, we categorized peak infection time points per viral family as the time category with the highest number of robustly changing genes, defined as genes with false discovery rate (FDR) < 0.05 and abs(effect size [ES]) > 0.1. Generally, peak DEG time point categories for each viral family were close to the peak MVS score time points demonstrated in Figure 1 (Figure 3B and Table S1).

(D–G) Association of MVS scores with the known risk factors of disease severity (D) vaccination status, (E) virus strain, (F) age of host, and (G) live virus across four different datasets from macaques infected with Machupo, influenza or Ebola virus. p value was determined by analysis of covariance (ANCOVA) test accounting for MVS score at pre-infection time point and a risk factor of interest as a covariate of the MVS score post infection. Transparent lines represent the linear connection between MVS scores from each individual macaque's healthy and infected time point, and the solid lines represent the summary line between healthy and infection MVS scores by group.

(H–J) UMAP visualization of 56,929 immune cells from 17 animals colored by (H) cell type, (I) day post infection (DPI), and (J) MVS score.

(K) Heatmap representing the average MVS score of each cell type across pre-infection and each day post infection, with values scaled by row. scRNA-seq data of whole blood from rhesus macaques infected with EBOV were collected at day 0 and multiple time points post infection. Asterisk values across figure are represented as follows: *p value < 0.05, **p value < 0.01, ***p value < 0.001, and ****p value < 0.0001. WB, whole blood; PBMC, peripheral blood mononuclear cell; MACV, Machupo virus; EBOV, Ebola virus; IFV, influenza virus.

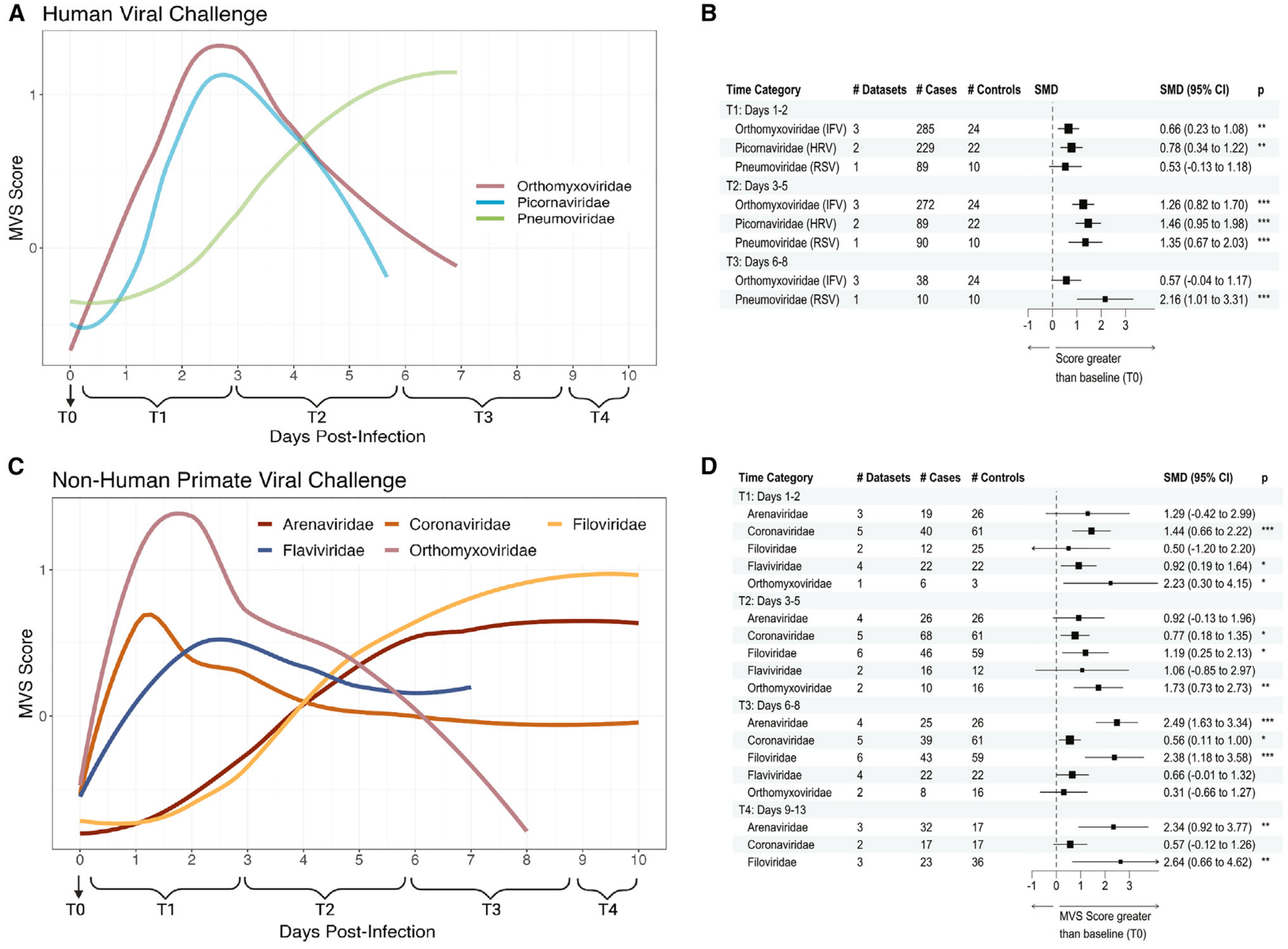

Figure 2. Longitudinal dynamics of the conserved antiviral response differ between viruses

(A and C) MVS scores across all datasets up to 10 days post infection across (A) 1,158 human and (C) 734 NHP challenge samples with time category annotated below with smoothed lines indicating the local regression (LOESS) curve fit by viral family.

(B and D) Forrest plot tables of the summary statistics generated for each viral infection in (B) human and (D) NHP challenge dataset by time category. Scores are calculated per sample as the difference between the geometric mean of the 161 overexpressed genes and 235 under-expressed genes in each signature and scaled within each dataset. Asterisk values across figure are represented as follows: *p value < 0.05, **p value < 0.01, ***p value < 0.001, and ****p value < 0.0001. SMD, standardized mean difference.

Next, we asked how conserved transcriptomic changes were across macaques between baseline and peak infection time points by viral family. We started by identifying gene signatures using our previously described multi-cohort analysis framework[20,21] to distinguish peak infection from the baseline T0 time point. We grouped the macaque datasets either by viral family—resulting in five signatures with one per viral family (Arena Sig., Corona Sig., Filo Sig., Flavi Sig., and Ortho Sig.; Table S7)—or with all 21 datasets together to create the viral response signature (VRS; Table S6). We compared each of these six newly developed signatures and the original MVS by gene to find limited overlap between the virus-specific signatures (ranging from 0 to 19 overlapping genes) (Figure 3C, left). Next, we performed enrichment analysis using the Blood Transcriptional Modules (BTMs), which are a set of modules defined on over 30,000 human blood transcriptomes derived from more than 500 studies available in public databases,[19] on the over- and under-expressed gene subsets from each signature separately (Table S8). There was much greater overlap in enriched modules between virus-specific signatures such that the Filo Sig. and Flavi Sig. overlapped with 8 pathways even though there were 0 overlapping genes (Figure 3D, right). Although some of the differences in genes may be due to variability in the statistical power, this result suggested that similar pathway networks are affected, even if driven by different genes (Figure 3C).

To further understand in what ways the signatures were connected, we further calculated a score for each BTM within the datasets grouped by viral families where the BTM was significant (p adj < 0.1; Figure 3D, track 2) and utilized links to connect BTMs across viral families where the BTM was enriched across the positive signature genes in both families. There were no connecting links across viruses by significant BTMs enriched in the negative signature gene subsets. Across all the viral families, there was a large number of upregulated pathways relating to myeloid and innate responses (Figure 3D). Similar analysis was also

Table 2. Time series analysis of MVS score by viral challenge in NHPs

Random effects:	Variance	Std. deviation	Corr	
Animal	0.054	0.232	–	–
Time	0.004	0.059	1.00	–
Residual	0.549	0.741	–	–
**Fixed effects:**	**β parameter estimate**	**std. error**	**t value**	**Pr(>|t|)**
Intercept	−0.372	0.190	−1.964	0.050
Time	0.912	0.209	4.361	1.55e–05
Time × time	−0.145	0.038	−3.852	1.32e–04
**Virus family**				
Arenaviridae	−0.507	0.235	−2.160	0.031
Coronaviridae	0.077	0.211	0.365	0.715
Filoviridae	−0.366	0.214	−1.712	0.088
Flaviviridae	−0.132	0.247	−0.533	0.594
**Virus family × time**				
Arenaviridae × time	−0.675	0.237	−2.852	0.005
Coronaviridae × time	−0.522	0.221	−2.368	0.018
Filoviridae × time	−0.754	0.228	−3.302	0.001
Flaviviridae × time	−0.516	0.245	−2.107	0.036
**Virus family × time × time**				
Arenaviridae × time × time	0.145	0.041	3.536	4.42e–04
Coronaviridae × time × time	0.096	0.039	2.451	0.015
Filoviridae × time × time	0.158	0.041	3.880	1.18e–04
Flaviviridae × time × time	0.105	0.042	2.471	0.014

Mixed-effects model used R package lmerTest. Comparisons are to *Orthmyxoviridae* viral challenge data. Data are from all NHPs infected with acute RNA virus, which includes 198 animals across 575 samples. Time points included were from day 0 to day 7 post-virus challenge.

performed across all the DEGs identified at peak time points from Figure 3B and demonstrated similar results (Figure S7). We also assessed the generalizability of each virus-specific signature to other viral families. All virus-specific signatures (Arena Sig., Corona Sig., Filo Sig., Flavi Sig., and Ortho Sig.; Table S7) distinguished healthy control and infected animals with high accuracy (AUROC >= 0.75; Figure 3E). By using this discovery/validation approach between viruses, this analysis demonstrates the robustly conserved innate responses that are upregulated across both hemorrhagic and nonhemorrhagic viral diseases.

To understand the whether the VRS was conserved across human virus infection, utilizing 3,183 human samples across 20 datasets of patients with one of 14 viral infections (Table S3), we found that the macaque VRS robustly distinguished viral infection from healthy across all symptomatic infections (Figures 3F and 3G). We separately looked at four human viral infections to demonstrate that the VRS signature robustly distinguished uninfected individuals from those infected with SARS-CoV-2, Ebola, or dengue virus (p adj < 0.0001). Notably, the VRS was conserved upon infection by the Chikungunya virus (p adj < 0.0001), an RNA virus whose family was not included in the VRS signature discovery data (Figure 3H).

Although VRS and MVS only had an overlap of 17 genes (Jaccard index [JI] = 0.03), we wanted to understand how similar the VRS and MVS responses were to each other. VRS and MVS scores were significantly positively correlated across macaque data ($r \geq 0.54$, $p < 1.4e–8$) and human data ($r \geq 0.42$, $p < 1.1e–8$) across all time points collected, although they were noticeably lower for *Flaviviridae* viral infection in macaques and *Filoviridae* infection in humans (Figures 3I and 3J). In BTM overrepresentation analysis of the macaque-derived VRS signature compared to the human-derived MVS signature, upregulated genes in both signatures were enriched in innate response and antiviral modules, whereas downregulated BTMs corresponded to adaptive responses, further demonstrating a conserved viral responses that transcends species and virus infections (Figure 3K and Table S9). Interestingly, while both signatures capture downregulation of genes associated with lymphoid cellular response modules, the negative genes represented in the VRS also capture non-lymphoid-specific responses such as signal transduction and eukaryotic initiation factor 3 (eIF3) pathways. Comparing the ability of the MVS versus the VRS to distinguish infection with viral families we have macaque data for, we see that while the MVS does a strong job at distinguishing respiratory viral infections (*Coronaviridae* and *Orthomyxoviridae*), the VRS robustly distinguishes *Flaviviridae* and *Filoviridae* infections—potentially because the VRS was trained on diverse viruses, whereas MVS was trained on only respiratory viruses (Figure S8). Together, these data validate macaques as robust models for studying human transcriptomic responses to viral infection.

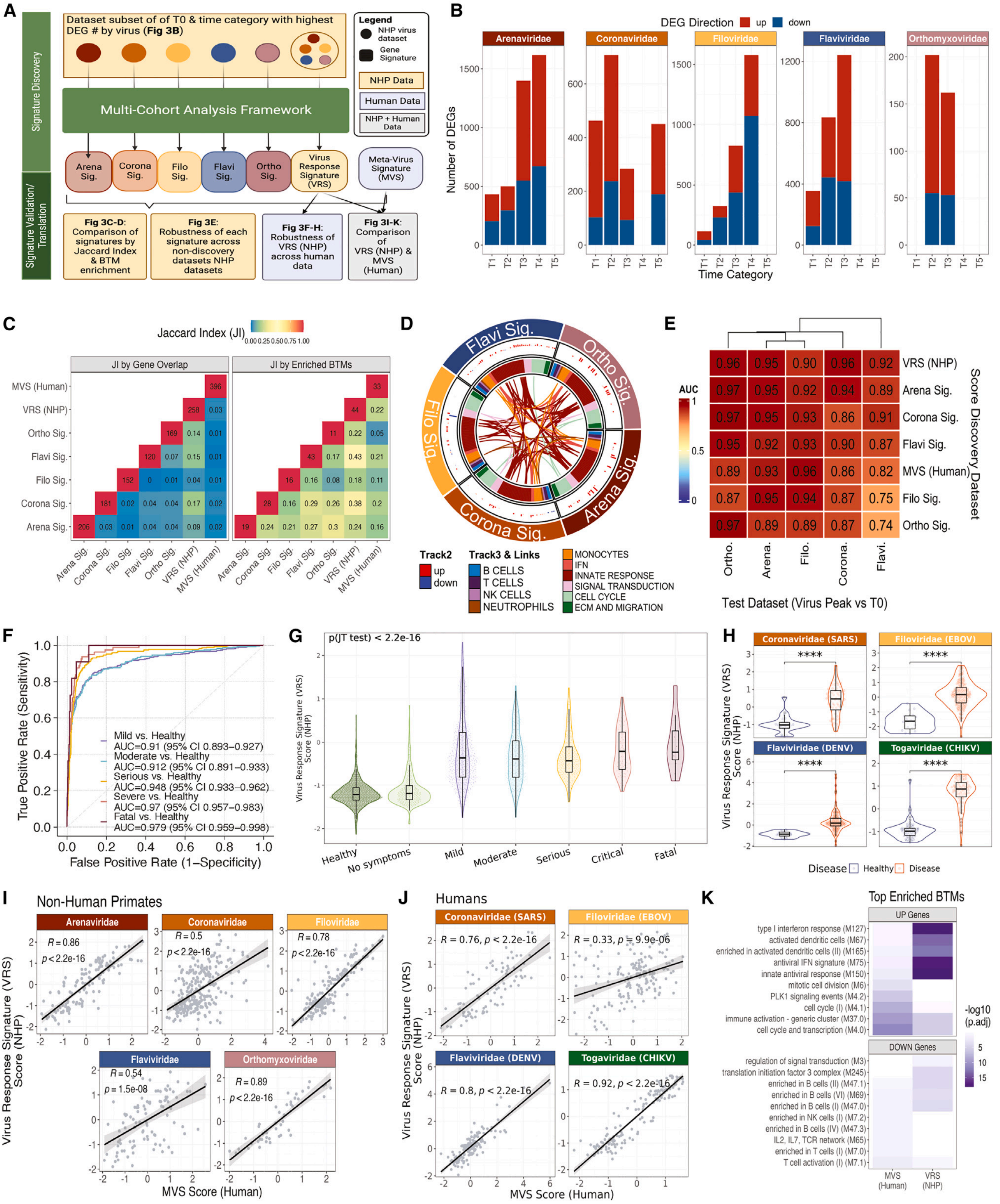

Figure 3. NHPs demonstrate virus-conserved responses to acute RNA viruses that robustly translate to humans

(A) Schematic of experimental design for (B)–(K).

(B) Significant DEGs at each time point category by viral family (effect size [ES] FDR < 0.05 and abs(ES) > 0.1).

(legend continued on next page)

Host response signature derived from macaques demonstrates robustness across acute and chronic viral infection in humans

Many patients have latent, chronic, or acute viral infections that are not caused by single-strand RNA (ssRNA) viruses. However, the VRS and the MVS were identified using only acute infections caused by ssRNA viruses. Therefore, we investigated whether immune responses in macaques and humans were conserved across diverse viruses and disease manifestations. We used the macaque-derived VRS to further investigate the generalizability, and subsequently its translatability, to a variety of human viral infections.

First, across acute infections, the VRS score was significantly higher ($p < 0.05$) in patients with adenovirus (a double-stranded DNA [dsDNA] virus), rotavirus (a double-stranded RNA [dsRNA] virus), Epstein-Barr virus (EBV; dsDNA virus), or human cytomegalovirus (HCMV; dsDNA virus) infections compared to healthy subjects (Figures 4A, 4B, 4C, and 4D). Second, we demonstrate that this response is robust in latent EBV (p adj < 0.01) but not in latent HCMV infection (Figures 4C and 4D). Third, the VRS was also significantly higher ($p < 0.01$) in patients with chronic HIV (RNA virus with reverse transcription [RT] step), hepatitis B (dsDNA virus with RT step), and hepatitis C (HCV; ssRNA virus) viral infection (Figures 4E, 4F, and 4G). Across the viruses studied here, the VRS demonstrated a stronger generalizability across represented viral infections in comparison to the MVS, potentially due to its discovery across a greater diversity of viral infections (Figure S9).

Consistently, the VRS distinguishes viral infection from a healthy state, and it even detects chronic infection, regardless of viral genome strandedness or nucleic acid intermediates. This shows that a shared host response exists in both macaques and humans across various types of viral infections, highlighting the power of macaque models for understanding human antiviral responses.

T cell responses differ across viral infections

Because the VRS and MVS showed different accuracy in distinguishing human infections by viral family, we next sought to understand the underlying mechanisms that might be driving differences in antiviral host responses (Figure S8). While overall innate and inflammation responses were conserved across both signatures, the VRS and MVS signatures differed in lymphoid response pathway gene representation (Figure 3K). Therefore, we hypothesized that lymphoid responses might be driving differences in virus-specific responses. To understand the mechanisms that might be driving these differences, we utilized the four gene modules that make up the MVS and that associate with different cell types and antiviral responses.[8]

To understand how these module responses changed with infection, we first compared the correlation of the VRS with each of the MVS modules and the overall MVS (Figure 5A). The overall MVS and three of its four modules showed the same correlational direction with VRS across each virus family. The exception was a dengue virus (DENV) infection dataset that showed a negative correlation between VRS and MVS and a different correlation direction between the VRS and Module 4, a lymphoid specific module. Module 4 also had differential responses in macaques infected with non-*Flaviviridae* viruses, in which Module 4 expression was inversely associated with the VRS score over time (Figures 5B and 5C), compared with *Flaviviridae* infections, in which Module 4 expression showed no consistent relationship with the VRS score.

To determine whether the DENV-specific difference in lymphoid responses was consistent across species, we examined the relationship between Module 4 and the VRS in humans. In humans infected with Chikagunya virus (CHIKV), DENV, Ebola virus (EBOV), or SARS-CoV-2, the negative correlation between module 4 and VRS was lower in EBOV ($r = -0.31$) and DENV infection ($r = -0.5$) compared to that of CHIV ($r = -0.8$) or SARS-CoV-2 infection ($r = -.61$; Figure 5D).

Because our previous work showed that genes in Module 4 were expressed higher in mild viral infections compared to severe viral disease, we further looked into the association between Module 4 and viral disease severity across these human datasets. We found that while Module 4 was significantly negatively associated with increased disease severity in SARS-CoV-2

(C) Jaccard similarity index of the signature genes between each signature, where annotation across the diagonal denotes the number of genes present in the signature, and all other annotations are the calculated Jaccard index (left). Jaccard similarity index of the blood transcription modules (BTMs) that contain one or more of the signature genes between each signature where annotation across the diagonal denoting same-score comparison is annotated with the number of BTMs represented by the signature, and all other annotations are the calculated Jaccard index.

(D) Circos plot of BTM enrichment analysis across positive signature genes by viral family. Each sector represents a viral family, and each point in all the tracks represents a BTM that was significant in at least one virus (p adj < 0.1). Track 2 is a barplot of the geometric mean of the effect sizes of the genes represented by each BTM that were generated from each virus-specific meta-analysis and plotted where the BTM was significant (p adj < 0.1). Each color in track 3 is a granular annotation for each BTM pathway. The inner track connects the same BTM across viral families if they are both (left) positively or (right) negatively enriched.

(E) Summary AUROC generated from the specific score (x axis) across the different viral family dataset subsets (y axis) comparing peak infection time category determined by (B) from healthy control animals.

(F) AUROC of human data using the NHP viral response signature (VRS) (n = 3,183).

(G) Combined violin and box and whisker plots of NHP VRS by viral severity of the samples from (F). Jonckheere-Terpstra (JT) trend test was used to assess the significance of the trend of the MVS score over severity.

(H) Violin plots of NHP VRS by virus and disease of the samples from (G).

(I) Spearman's correlation between the calculated MVS score and the generated NHP VRS. Each dot is a single blood sample from an NHP across all time points collected (743 samples).

(J) Spearman's correlation between the calculated MVS (human) score and the generated VRS (NHP) score. Each dot is a single blood sample from various public human gene expression datasets (n = 638).

(K) Comparison of signature-enriched BTM pathways in the overexpressed (UP genes) and under-expressed (DOWN genes) genes in the MVS (human) and VRS (NHP) pathways. Top 5 pathways (ordered by p adj) were chosen per signature's up and down genes. Asterisk values across figure are represented as follows: *p value < 0.05, **p value < 0.01, ***p value < 0.001, and ****p value < 0.0001.

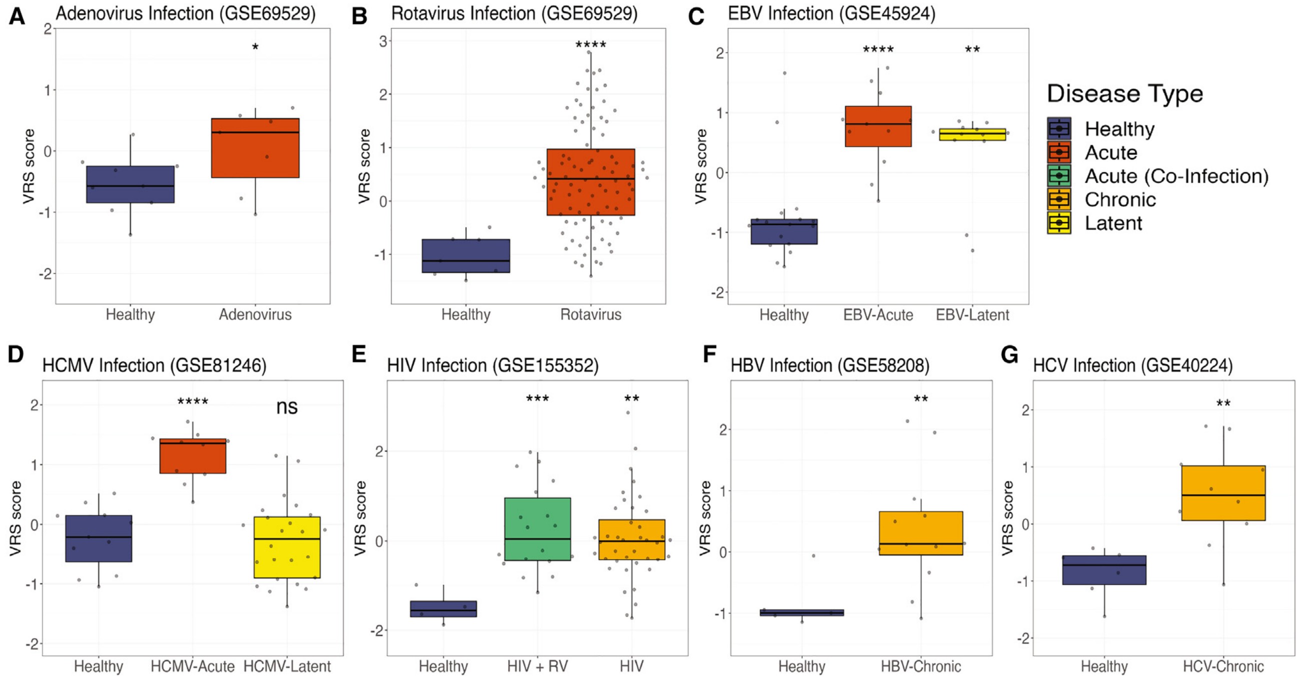

Figure 4. Macaque-discovered antiviral response is consistent in human acute and chronic but not latent viral infections

VRS score in blood samples from healthy control subjects versus patients with (A) adenovirus infection, (B) rotavirus infection, (C) acute or latent EBV infection, (D) acute or latent HCMV infection, (E) HIV infection or HIV co-infection with a respiratory virus (RV), (F) chronic HBV infection, and (G) chronic HCV infection. Data presented as box and whisker plots. Significance values were determined using an unpaired, one-sided Wilcoxon ranked-sum test looking at whether healthy VRS scores are less than comparator group VRS scores. Bonferroni correction for multiple hypothesis testing was applied per subfigure, and significance values were assigned by asterisk. Asterisk values across figure are represented as follows: *p value < 0.05, **p value < 0.01, ***p value < 0.001, and ****p value < 0.0001.

and CHIKV infection ($p <= 3.7e–10$), the association was not significant in DENV disease severity ($p = 0.12$) (Figures 5E and 5F). Together, these data suggest a potential difference in lymphoid responses to different viral infections that are conserved across species.

To understand which cells drive the difference in lymphoid cellular responses, we identified two scRNA-seq PBMC datasets with healthy controls and patients with either SARS-CoV-2 or dengue virus infection (Figures S10A– S10C).[22,23] We focused on patients with severe disease manifestations because the strongest differences in Module 4 were demonstrated to be associated with severity.[8] We found that the T and NK cell populations had higher expression of the genes in Module 4 in patients with severe disease (Figures 5G and 5H). DEG analysis of these cell types found differences in the CD8 T cell responses between severe COVID-19 and dengue disease (Figure 5H and Table S10). Specifically, compared to healthy controls, genes marking effector and cytotoxic CD8 T cell profiles (*GZMB*, *NKG7*, *GZMH*, *PRF1*, and *CCL5*) were upregulated in severe dengue and downregulated in severe COVID-19 (Figure 5H). Further, BTM enrichment analysis using DEGs that were identified at a per-virus-infected-patient level in comparison to healthy controls showed that generally severe dengue patients upregulated genes related to T cell activation and differentiation modules, whereas severe COVID-19 patients downregulated these module genes (Figure 5I). A similar trend was present in CD4 T cell responses (Figures S10D and S10E and Table S10); however, there were no strong differences in NK cell responses between severe COVID-19 and dengue disease (Figures S10F and S10G). This scRNA-seq analysis suggests a divergence in lymphoid responses between COVID-19 and DENV that may be linked to CD8 T effector functions and that may be important to address in vaccination strategies toward these different viruses.

DISCUSSION

Emerging and reemerging viral diseases remain a constant global health threat mandating the development of new solutions to combat future epidemics and pandemics. Multiple viruses of public health concern are understudied in humans; however, macaques remain an established model for understanding human disease with numerous independent virus studies published in these models to provide a wealth of information on viral disease. Here, we report that macaques are a reliable model of human antiviral transcriptomic responses. Our results also extended the generalizability of virus- and species-conserved antiviral responses to several acute RNA viral infections of WHO priority concern, which include highly lethal viruses for which human data does not exist, and to viral infections by DNA viral infections and chronic viral diseases. Notably, we identified differences in longitudinal dynamics of antiviral responses and T cell functions depending on the infecting virus. Together, these data provide detailed insights into the dynamic immune

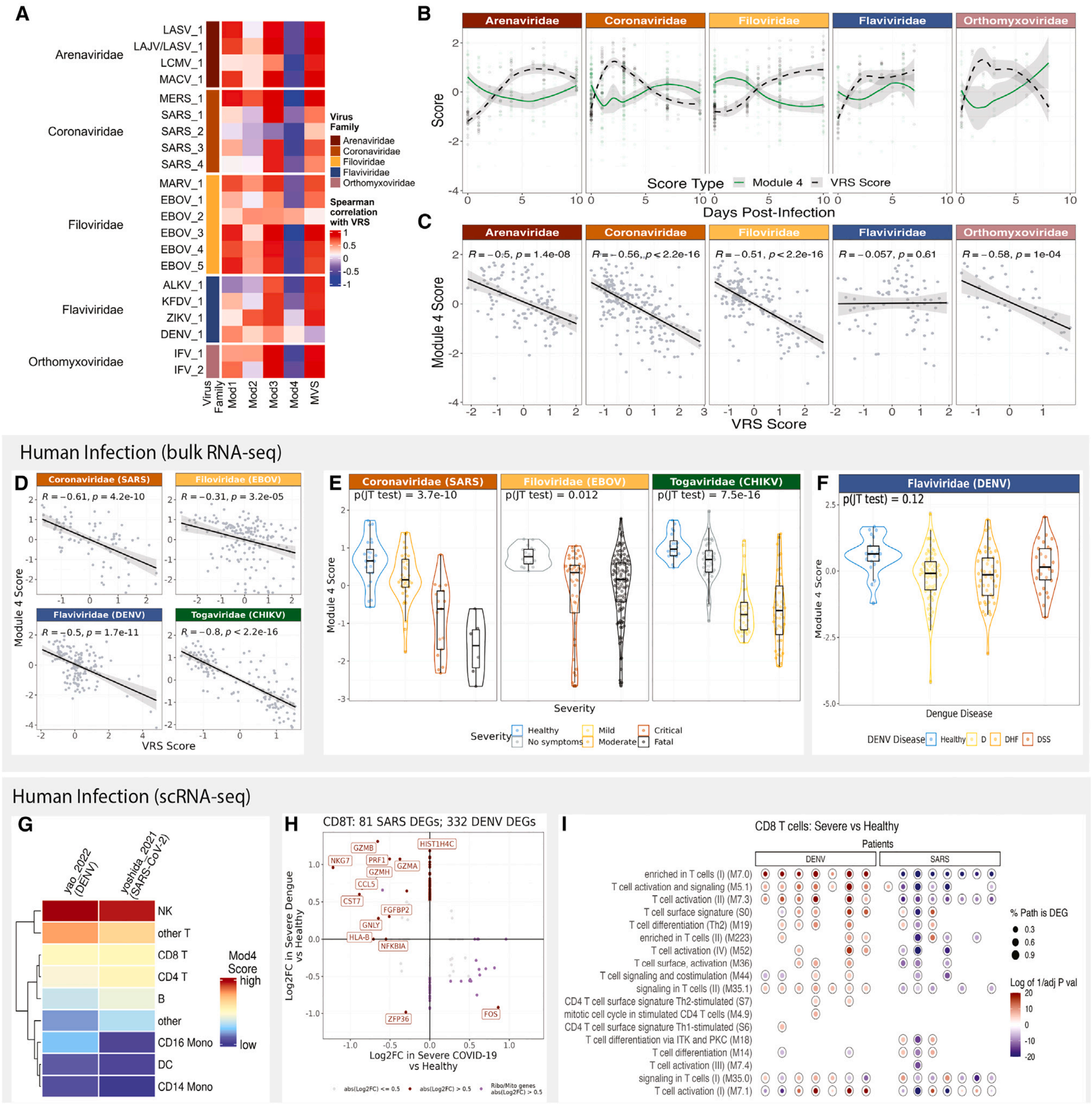

Figure 5. T cell responses differ between viruses in macaque and human viral infection

(A) Distribution of the Module 4 scores across macaques, comparing uninfected, healthy macaques to those at peak MVS score by viruses across 5 viral families. Each point represents a blood sample. Significance values were determined using an unpaired Wilcoxon ranked-sum test with Bonferroni correction for multiple hypothesis testing and assigned by asterisk.

(B) Comparison of Module 4 scores to VRS scores across time in 4 viral families.

(C) Comparison of Module 4 scores to VRS scores across the 4 viral species collected within the *Flaviviridae* family.

(D) Module 4 score to VRS score in human data across 4 viral infections.

(E) Module 4 scores by viral severity across CHIKV, EBOV, and SARS-CoV-2 viral infection. p values were computed using JT trend test. Data presented as combined violin and box and whisker plots.

(F) Module 4 scores across disease time point and dengue disease type. p values were computed using JT trend test.

(G) Expression of Module 4 scores by cell type in 2 scRNA-seq datasets.

(legend continued on next page)

landscape of viral diseases across species that can provide potential targets for host-directed immunomodulatory antivirals.

Despite the MVS being discovered using respiratory viral infections in humans, we show that this response extends to macaques and generalizes to *Flaviviridae* and *Arenaviridae* infections. Interestingly, we show that the longitudinal kinetics of its induction vary by viral family. Our results are consistent with reported differences in incubation periods of these viruses in natural human disease: SARS-CoV-2, influenza, and HRV infection show shortened incubation periods relative to Ebola, Lassa, and RSV.[24–26] Hypotheses for these kinetic differences in response include the following: differences in host sensing of the virus (e.g., via pathogen recognition receptors), variability in virus replication dynamics, virus-specific proteins that evade/induce host responses, differences in infection route and/or tissue tropism, and virus latency.[27,28] Interestingly, *Arenaviridae*, *Filoviridae*, and RSV are negative-stranded RNA viruses (–ssRNA) that replicate in the cytoplasm and demonstrate longer periods of MVS induction compared to the MVS kinetics in infection by influenza (–ssRNA, nucleus replicating) and positive-stranded RNA viruses (+ssRNA) such as *Coronaviridae* and *Flaviviridae* viruses. This is not unexpected as studies have shown that genome type may impact replication dynamics and associated host inflammation.[29–31] While our study focuses on cells in the blood, there could be differences in the types of tissues infected and the magnitude of cytokine and ISG production, resident cell activation, and immune cell recruitment detectable in peripheral blood. Further studies on comparative viral immunology and disease are required to ascertain the drivers of these different dynamics.

We further define a new virus-conserved gene signature identified across viral infection of macaques, VRS, that is robust across infection of diverse macaque and human populations. In this study, the VRS distinguished virus infection across a greater range of viruses than the MVS, with its range spanning the Baltimore Virus Classification system (here we include six out of seven Baltimore Classification groups). However, the MVS does a stronger job in distinguishing infection by *Coronaviridae* and *Orthomyxoviridae* viruses, while the VRS does a stronger job with *Flaviviridae* and *Filoviridae* virus infection in human data. These differences are likely driven by differences in the diseases included in the discovery datasets used for signature creation, as the MVS was solely respiratory viruses, whereas the MVS included lethal, hemorrhagic fever diseases. This is also reflected in the gene composition of each signature. While both signatures capture the increase in innate immune response genes and downregulation in lymphoid response genes, the underrepresented genes in the VRS signature are also enriched in signal transduction and eukaryotic initiation factor (eIF3) pathways. Viruses and associated host responses are known to manipulate and alter cellular signal transduction pathways.[32] Additionally, many viruses have been shown to recruit and/or directly bind to eIF3—a protein complex important to translation initiation—that include Ebola, rabies and hepatitis C viruses.[33–36] From the host response side, interferon-induced protein with tetratricopeptide repeats (IFIT) has been shown to inhibit HCV replication via eIF3 targeting.[36,37] Together, these data suggest the importance of conducting studies across diverse virus infections based upon limitations in viral disease diversity and the added utility of available macaque data as an added source of data.

Substantial overlap between MVS and VRS demonstrates that human antiviral responses translate to macaques, and vice versa, suggesting that there are evolutionarily conserved antiviral response pathways that play important roles across a broad range of viruses. Type I IFNs are an ancient and conserved set of inflammatory cytokines important to the first-line defense against viruses across the evolution of mammals.[38,39] The host-pathogen arms race, in which there is evolutionary pressure for viruses to evade the host immune response and for the host to keep up a strong antiviral response, is responsible for the complexity of our immune system.[40–42] In fact, many genes in mammalian IFN pathways (e.g., cGAS, OASs, STING, and MAVS) show positive natural selection, which may be driven by the plethora of viruses known to interact and/or inhibit these genes (e.g., Flavivirus targeting of STING, HCV targeting of MAVS).[43–47] It has been hypothesized that redundancy of ISGs allows for these genes to tolerate more mutation, remaining a reliable antiviral response system.[43] Therefore, it is not unexpected that many of the pathways represented in both the VRS and MVS are attributed to myeloid cells and relate to IFN responses, since myeloid cells are early responders to viral infection, major producers of type I IFN and ISGs, and promote inflammation and promote killing of infected cells.[48] As infection continues, a myeloid bias during hematopoiesis can produce more of these effector cells to battle the infection, but myeloid cells can also take on immunosuppressive roles later in infection.[48] Thus, both the myeloid cell numbers and their associated antiviral phenotypes are important and dynamic across the course of infection, which is represented in the species-conserved MVS and VRS genes.

The virus-conserved nature of this response (i.e., across viruses with diverse genomes) is consistent with previous work suggesting there is crosstalk between DNA and RNA sensors.[49] For example, the cGAS-STING axis, established as a cytosolic DNA sensing pathway, has been involved in RNA viral infections and also shown to activate cytosolic RNA sensors via STING binding to RIG-I/MAVS (cytosolic RNA sensors).[50–55] Downstream, these pathways drive robust IFN induction and inflammation. Interestingly, the conserved response was present in chronic virus disease. Studies have shown that there are changes in the host immune response upon chronic infection that lead to low levels of type I IFN, which may drive associated downstream inflammation as well as changes to T cell functioning that are detectable by the VRS.[56]

In the context of latent infections, VRS could significantly distinguish latent EBV but not latent HCMV infection from healthy controls. Unlike in chronic infection where the virus is still present

(H) Differential gene expression analysis of CD8 T cells across scRNA-seq data from COVID-19 and dengue patients between patients with severe disease compared to healthy controls.

(I) BTM enrichment analysis of differentially expressed genes from each severe patient compared to the dataset's healthy patients. Color of p adj value was determined by whether pathway analysis was performed on the upregulated genes (red) or the downregulated genes (blue).

and replicating, albeit at low levels, in viral latency, the virus does not replicate and aims to avoid host detection. However, there is evidence for that latent EBV can still drive inflammation.[57,58] It is also possible that the differences in between the EBV cohort (GSE45924_GPL6883) and the HCMV cohort (GSE81246) may contribute to differences in VRS detection.[59,60] The EBV cohort was a prospective cohort following EBV-naive college undergraduate students, a younger population. In contrast, the HCMV cohort was a cross-sectional observational study with a mean age of 38 years (20–67) years and unknown date of CMV acquisition. Note that B cells are the primary target of EBV infection, whereas epithelial cells, endothelial cells, fibroblasts, and smooth muscle cells are the primary targets of HCMV, which could lead to differences in the VRS (and MVS) expression detected in PBMCs/whole blood and thereby affect our ability to distinguish between latent and uninfected samples. Further studies are needed to understand conserved host response kinetics in latent and reactivated infection.

Our data suggest that while T cells are crucial for mounting effective immune responses during viral infections, differences in T cell transcriptional responses between viruses exist and could contribute to differential disease outcome. During COVID-19, lymphopenia and exhausted T cells are found in patients with severe and fatal disease outcomes and may serve as a potential prognostic for disease outcome.[61,62] Lymphopenia has also been described in severe Ebola infections, while exhausted T cells have also been a marker of chronic viral infections such as HIV, hepatitis B, and hepatitis C.[63,64] The role of T cell responses in dengue has been highly disputed. The majority of symptomatic dengue disease is driven by secondary dengue infections, yet it is unclear whether pre-existing dengue-specific T cells play a protective or pathogenic role. Generally, studies consistently report an expansion of pre-existing T cell populations readily activated upon secondary dengue infection,[65] which is in line with our data. However, while some studies report no transcriptional differences in the quantity and quality of DENV-specific CD4 and CD8 T cell populations by disease severity,[66,67] others suggest that the expansion of pre-existing cross-reactive T cells drives severity through ineffective viral control and aberrant cytokine responses.[68–70] Though we cannot distinguish DENV specificity or pathologic versus protective functional differences, we do see a general activation of the T cell compartment. Interestingly, all macaques had a primary flavivirus infection and consistently demonstrated differing trends in the expression of MVS genes relating to B, T, and NK cell responses (Module 4) compared to other viral infections. This result suggests *Flaviviridae*-specific differences in T cell response induction independent of prior exposure. These data together are important because they highlight that general T cell activation and proliferation may not directly equate to protective viral responses and suggest further work is needed in dissecting specific T cell subsets and their role across viral disease pathogenicity.

In conclusion, we conducted a comprehensive analysis of host responses to a wide range of viruses, using transcriptomic data from both human and macaque cohorts. Leveraging macaque data allowed us to gather pre-symptomatic, post-inoculation time points from infection by pathogenic and lethal viruses that are otherwise very difficult, if not impossible, to obtain from humans. Our integrated analysis across heterogeneous macaque and human cohorts identified highly generalizable antiviral responses conserved in acute and chronic viral disease. Moreover, our analyses identified differences in the longitudinal dynamics of host response induction and resolution by viral family. These results further support the reliability of macaque models in studying human antiviral responses and are useful for pandemic preparedness. Specifically, our work identifies several key areas for future research and development of antiviral countermeasures, including the design of new intervention strategies such as diagnostics and therapeutic timing, the optimization of macaque challenge study design for emerging and reemerging viruses, and the development of broad-spectrum host-directed immunomodulatory therapies. These findings underscore the importance of continued comparative research across transcriptomic responses to diverse viruses in macaques and humans, with the ultimate goal of improving our ability to predict, prevent, and treat viral infections.

Limitations of the study

Our study has several potential limitations. First, in a number of microarray datasets, not all genes in each gene set tested were measured, in which case, we used the subset of genes in the particular dataset. Our previous work has shown that many of the genes within the MVS score are highly correlated, and only a subset is required to detect conserved responses. Second, all macaque genes were converted to human homologs, ignoring the expression of macaque genes that do not have a clear human homolog and/or may be important to overall antiviral response dynamics. Third, we only analyzed transcriptome data from blood samples. However, differences in antiviral responses may occur at the tissue level and site of infection that we did not analyze. Fourth, some viral families we analyzed only had a small representation of viral species and did not have different viral variants accounted for. While we use grouping at the viral family level to identify conserved patterns, these may not be applicable to every virus within that viral family. For example, not all viruses cause symptomatic disease in both macaques and humans, such as HIV in humans and simian immunodeficiency virus in macaques. While we included all macaque viral datasets available and a broad range of viruses with epidemic and pandemic concerns, the MVS and VRS responses need to continually be tested in new viral infection datasets. Ideally, we would have compared scRNA-seq responses to the same virus between humans and macaques. However, we were unable to identify appropriate scRNA-seq data to perform such a comparison as these datasets are limited.[71] Thus, we compared human COVID-19 data with macaque Ebola data because we have already shown that the MVS is conserved across 16 different viruses, including COVID-19 and Ebola in humans,[8] and also conserved across viruses in macaques.

STAR★METHODS

Detailed methods are provided in the online version of this paper and include the following:

- KEY RESOURCES TABLE
- RESOURCE AVAILABILITY

- Lead contact
- Materials availability
- Data and code availability
- METHOD DETAILS
- QUANTIFICATION AND STATISTICAL ANALYSIS
  - Non-human primate dataset collection and preprocessing
  - Human dataset collection and preprocessing
  - Comparison of baseline macaque gene expression
  - Gene signatures and scoring
  - Mixed-effects model for time point data
  - Gene set overrepresentation enrichment analysis
  - Analysis of single-cell RNA sequencing
  - Figure generation

SUPPLEMENTAL INFORMATION

Supplemental information can be found online at https://doi.org/10.1016/j.celrep.2024.113706.

ACKNOWLEDGMENTS

We are grateful to all the labs that conducted the experiments and generated and publicly shared their data. We also thank all study participants and their families for being involved in all included studies. We thank Dylan Kotliar for sharing processed scRNA-seq data with us. Finally, we thank the members of the Blish Lab and Khatri Lab for useful suggestions: Denis Dermadi, Maïgane Diop, Mike Freedman, Ananthakrishnan Ganesan, Umay Geyikci, Sanjana Gupta, Rebecca Hamlin, Larry Kalesinskas, Ian Lee, Maddie Lee, Michelle Leong, Yiran Liu, Giovanny Martinez-Colón, Andrew Moore, Ruoxi Pi, Kass Pinedo, Thanmayi Ranganath, Izumi de los Rios Kobrara, Makeda Robinson, Sonieda Rodriguez, Arjun Rustagi, Sarah Sackey, Marion Santo, Ben Solomon, Mikayla Stabile, Simone Thair, Aaron Wilk, and Mengyang Zhang. K.R. is funded by the National Science Foundation Graduate Research Fellowship 2019282939 and Bio-X graduate Stanford Graduate Fellowship. J.T. is funded by National Science Scholarship (PhD) from the Agency for Science, Technology and Research (A*STAR), Singapore. Z.Y. was supported by a Thrasher Research Fund early career award program grant and by a postdoctoral fellowship from the Maternal & Child Health Research Institute, Lucile Packard Foundation for Children's Health. V.D. was supported by a Chan Zuckerberg Biohub Collaborative Postdoctoral Fellowship. S.E. is funded by a NIAID grant RO1AI158569, an Investigator Initiated Award number W81XWH2210283 and an expansion award number W81XWH2110456 from the DoD office of the CDMRP/PRMRP, and a Defense Threat Reduction Fundamental Research to Counter Weapons of Mass Destruction grant HDTRA11810039. S.E. and C.A.B. are Investigators of the Chan Zuckerberg Biohub. C.A.B. is funded in part by NIH DP1 DA046089, a 2019 Sentinel Pilot Project from the Bill and Melinda Gates Foundation, and OP113682 and U19AI057229 from the Bill and Melinda Gates Foundation. P.K. is funded in part by the Bill and Melinda Gates Foundation (OPP1113682); the National Institute of Allergy and Infectious Diseases (NIAID) grants 1U19AI109662, U19AI057229, and 5R01AI125197; and Department of Defense contract W81XWH-18-1-0253. S.E. and P.K. are funded in part by Department of Defense contract W81XWH1910235 and the Ralph & Marian Falk Medical Research Trust. The funders had no role in study design, data collection and analysis, decision to publish, or preparation of the manuscript.

AUTHOR CONTRIBUTIONS

K.R., C.A.B., and P.K. conceived the study. C.A.B. and P.K. supervised the study. K.R., H.Z., J.T., Z.Y., and M.D. collected, annotated, processed, and analyzed data. K.R., C.A.B., and P.K. interpreted analysis results and wrote the manuscript. S.E. supervised the dengue scRNA-seq study cohort. V.D. and S.E. designed study and enrolled patients with dengue infection. Z.Y. profiled and processed single-cell RNA-seq of PBMCs from patients with dengue infection. M.K., M.R., J.-P.M.T., M.G., K.E.F., J.R.F., K.S.C., D.C.D., and R.A.S. designed and carried out the study profiling RNA-seq of PBMCs from NHPs with SARS-CoV-2 infection.

DECLARATION OF INTERESTS

The authors declare no competing interests.

Received: July 3, 2023
Revised: November 2, 2023
Accepted: January 9, 2024
Published: January 30, 2024

REFERENCES

1. Rosenberg, R. (2015). Detecting the emergence of novel, zoonotic viruses pathogenic to humans. Cell. Mol. Life Sci. *72*, 1115–1125.
2. Carrasco-Hernandez, R., Jácome, R., López Vidal, Y., and Ponce de León, S. (2017). Are RNA Viruses Candidate Agents for the Next Global Pandemic? A Review. ILAR J. *58*, 343–358.
3. Morens, D.M., and Fauci, A.S. (2020). Emerging Pandemic Diseases: How We Got to COVID-19. Cell *182*, 1077–1092.
4. Kilpatrick, A.M., and Randolph, S.E. (2012). Drivers, dynamics, and control of emerging vector-borne zoonotic diseases. Lancet *380*, 1946–1955.
5. Domingo, E., and Holland, J.J. (1997). RNA VIRUS MUTATIONS AND FITNESS FOR SURVIVAL. Annu. Rev. Microbiol. *51*, 151–178.
6. Estes, J.D., Wong, S.W., and Brenchley, J.M. (2018). Nonhuman primate models of human viral infections. Nat. Rev. Immunol. *18*, 390–404.
7. Andres-Terre, M., McGuire, H.M., Pouliot, Y., Bongen, E., Sweeney, T.E., Tato, C.M., and Khatri, P. (2015). Integrated, Multi-cohort Analysis Identifies Conserved Transcriptional Signatures across Multiple Respiratory Viruses. Immunity *43*, 1199–1211.
8. Zheng, H., Rao, A.M., Dermadi, D., Toh, J., Murphy Jones, L., Donato, M., Liu, Y., Su, Y., Dai, C.L., Kornilov, S.A., et al. (2021). Multi-cohort analysis of host immune response identifies conserved protective and detrimental modules associated with severity across viruses. Immunity *54*, 753–768.e5.
9. Prioritizing Diseases for Research and Development in Emergency Contexts https://www.who.int/activities/prioritizing-diseases-for-research-and-development-in-emergency-contexts
10. Reyes, M., Filbin, M.R., Bhattacharyya, R.P., Sonny, A., Mehta, A., Billman, K., Kays, K.R., Pinilla-Vera, M., Benson, M.E., Cosimi, L.A., et al. (2021). Plasma from patients with bacterial sepsis or severe COVID-19 induces suppressive myeloid cell production from hematopoietic progenitors in vitro. Sci. Transl. Med. *13*, eabe9599.
11. Reynard, S., Carnec, X., Picard, C., Borges-Cardoso, V., Journeaux, A., Mateo, M., Germain, C., Hortion, J., Albrecht, L., Perthame, E., et al. (2023). A MOPEVAC multivalent vaccine induces sterile protection against New World arenaviruses in non-human primates. Nat. Microbiol. *8*, 64–76.
12. Marzi, A., Feldmann, F., Hanley, P.W., Scott, D.P., Günther, S., and Feldmann, H. (2015). Delayed Disease Progression in Cynomolgus Macaques Infected with Ebola Virus Makona Strain. Emerg. Infect. Dis. *21*, 1777–1783.
13. Maroney, K.J., Pinski, A.N., Marzi, A., and Messaoudi, I. (2021). Transcriptional Analysis of Infection With Early or Late Isolates From the 2013–2016 West Africa Ebola Virus Epidemic Does Not Suggest Attenuated Pathogenicity as a Result of Genetic Variation. Front. Microbiol. *12*, 714817.
14. Fukuyama, S., Iwatsuki-Horimoto, K., Kiso, M., Nakajima, N., Gregg, R.W., Katsura, H., Tomita, Y., Maemura, T., da Silva Lopes, T.J.,

CellPress
OPEN ACCESS

Watanabe, T., et al. (2020). Pathogenesis of Influenza A(H7N9) Virus in Aged Nonhuman Primates. J. Infect. Dis. *222*, 1155–1164.

15. Skinner, J.A., Zurawski, S.M., Sugimoto, C., Vinet-Oliphant, H., Vinod, P., Xue, Y., Russell-Lodrigue, K., Albrecht, R.A., García-Sastre, A., Salazar, A.M., et al. (2014). Immunologic characterization of a rhesus macaque H1N1 challenge model for candidate influenza virus vaccine assessment. Clin. Vaccine Immunol. *21*, 1668–1680.
16. Kotliar, D., Lin, A.E., Logue, J., Hughes, T.K., Khoury, N.M., Raju, S.S., Wadsworth, M.H., Chen, H., Kurtz, J.R., Dighero-Kemp, B., et al. (2020). Single-Cell Profiling of Ebola Virus Disease In Vivo Reveals Viral and Host Dynamics. Cell *183*, 1383–1401.e19.
17. Koushki, K., Salemi, M., Miri, S.M., Arjeini, Y., Keshavarz, M., and Ghaemi, A. (2021). Role of myeloid-derived suppressor cells in viral respiratory infections; Hints for discovering therapeutic targets for COVID-19. Biomed. Pharmacother. *144*, 112346.
18. Wilk, A.J., Lee, M.J., Wei, B., Parks, B., Pi, R., Martínez-Colón, G.J., Ranganath, T., Zhao, N.Q., Taylor, S., Becker, W., et al. (2021). Multi-omic profiling reveals widespread dysregulation of innate immunity and hematopoiesis in COVID-19. J. Exp. Med. *218*, e20210582.
19. Li, S., Rouphael, N., Duraisingham, S., Romero-Steiner, S., Presnell, S., Davis, C., Schmidt, D.S., Johnson, S.E., Milton, A., Rajam, G., et al. (2014). Molecular signatures of antibody responses derived from a systems biological study of 5 human vaccines. Nat. Immunol. *15*, 195–204.
20. Sweeney, T.E., Haynes, W.A., Vallania, F., Ioannidis, J.P., and Khatri, P. (2017). Methods to increase reproducibility in differential gene expression via meta-analysis. Nucleic Acids Res. *45*, e1.
21. HAYNES, W.A., VALLANIA, F., LIU, C., BONGEN, E., TOMCZAK, A., Andres-Terrè, M., LOFGREN, S., TAM, A., DEISSEROTH, C.A., LI, M.D., et al. (2017). EMPOWERING MULTI-COHORT GENE EXPRESSION ANALYSIS TO INCREASE REPRODUCIBILITY. Biocomput *22*, 144–153.
22. Yoshida, M., Worlock, K.B., Huang, N., Lindeboom, R.G.H., Butler, C.R., Kumasaka, N., Dominguez Conde, C., Mamanova, L., Bolt, L., Richardson, L., et al. (2022). Local and systemic responses to SARS-CoV-2 infection in children and adults. Nature *602*, 321–327.
23. Ghita, L., Yao, Z., Xie, Y., Duran, V., Cagirici, H.B., Samir, J., Osman, I., Rebellón-Sánchez, D.E., Agudelo-Rojas, O.L., Sanz, A.M., et al. (2023). Global and cell type-specific immunological hallmarks of severe dengue progression identified via a systems immunology approach. Nat. Immunol. *24*, 2150–2163.
24. Lessler, J., Reich, N.G., Brookmeyer, R., Perl, T.M., Nelson, K.E., and Cummings, D.A.T. (2009). Incubation periods of acute respiratory viral infections: a systematic review. Lancet Infect. Dis. *9*, 291–300.
25. Eichner, M., Dowell, S.F., and Firese, N. (2011). Incubation Period of Ebola Hemorrhagic Virus Subtype Zaire. Osong Public Health Res. Perspect. *2*, 3–7.
26. Holmes, G.P., McCormick, J.B., Trock, S.C., Chase, R.A., Lewis, S.M., Mason, C.A., Hall, P.A., Brammer, L.S., Perez-Oronoz, G.I., McDonnell, M.K., et al. (1990). Lassa Fever in the United States. N. Engl. J. Med. *323*, 1120–1123.
27. Carrat, F., Vergu, E., Ferguson, N.M., Lemaitre, M., Cauchemez, S., Leach, S., and Valleron, A.-J. (2008). Time Lines of Infection and Disease in Human Influenza: A Review of Volunteer Challenge Studies. Am. J. Epidemiol. *167*, 775–785.
28. Ebola Disease - Diagnosis https://www.cdc.gov/vhf/ebola/diagnosis/index.html#:∼:text=Ebola%20virus%20can%20be%20detected,low%20levels%20of%20Ebola%20virus.
29. Yoneyama, M., Kikuchi, M., Natsukawa, T., Shinobu, N., Imaizumi, T., Miyagishi, M., Taira, K., Akira, S., and Fujita, T. (2004). The RNA helicase RIG-I has an essential function in double-stranded RNA-induced innate antiviral responses. Nat. Immunol. *5*, 730–737.
30. Andrejeva, J., Childs, K.S., Young, D.F., Carlos, T.S., Stock, N., Goodbourn, S., and Randall, R.E. (2004). The V proteins of paramyxoviruses bind the IFN-inducible RNA helicase, mda-5, and inhibit its activation of the IFN-beta promoter. P Natl Acad Sci Usa *101*, 17264–17269.
31. Ramos, H.J., and Gale, M. (2011). RIG-I like receptors and their signaling crosstalk in the regulation of antiviral immunity. Curr. Opin. Virol. *1*, 167–176.
32. Moens, U. (2022). Role of Signaling Pathways in the Viral Life Cycle 2.0. Int. J. Mol. Sci. *23*, 7857.
33. Komarova, A.V., Real, E., Borman, A.M., Brocard, M., England, P., Tordo, N., Hershey, J.W.B., Kean, K.M., and Jacob, Y. (2007). Rabies virus matrix protein interplay with eIF3, new insights into rabies virus pathogenesis. Nucleic Acids Res. *35*, 1522–1532.
34. Daughenbaugh, K.F., Fraser, C.S., Hershey, J.W.B., and Hardy, M.E. (2003). The genome-linked protein VPg of the Norwalk virus binds eIF3, suggesting its role in translation initiation complex recruitment. EMBO J. *22*, 2852–2859.
35. Le Sage, V., Cinti, A., McCarthy, S., Amorim, R., Rao, S., Daino, G.L., Tramontano, E., Branch, D.R., and Mouland, A.J. (2017). Ebola virus VP35 blocks stress granule assembly. Virology *502*, 73–83.
36. Wang, C., Pflugheber, J., Sumpter, R., Sodora, D.L., Hui, D., Sen, G.C., and Gale, M. (2003). Alpha Interferon Induces Distinct Translational Control Programs To Suppress Hepatitis C Virus RNA Replication. J. Virol. *77*, 3898–3912.
37. Diamond, M.S., and Farzan, M. (2013). The broad-spectrum antiviral functions of IFIT and IFITM proteins. Nat. Rev. Immunol. *13*, 46–57.
38. Pestka, S. (2007). The Interferons: 50 Years after Their Discovery, There Is Much More to Learn. J. Biol. Chem. *282*, 20047–20051.
39. McNab, F., Mayer-Barber, K., Sher, A., Wack, A., and O'Garra, A. (2015). Type I interferons in infectious disease. Nat. Rev. Immunol. *15*, 87–103.
40. Fensterl, V., Chattopadhyay, S., and Sen, G.C. (2015). No Love Lost Between Viruses and Interferons. Annu. Rev. Virol. *2*, 549–572.
41. García-Sastre, A. (2017). Ten Strategies of Interferon Evasion by Viruses. Cell Host Microbe *22*, 176–184.
42. Duggal, N.K., and Emerman, M. (2012). Evolutionary conflicts between viruses and restriction factors shape immunity. Nat. Rev. Immunol. *12*, 687–695.
43. Judd, E.N., Gilchrist, A.R., Meyerson, N.R., and Sawyer, S.L. (2021). Positive natural selection in primate genes of the type I interferon response. BMC Ecol. Evol. *21*, 65.
44. Hancks, D.C., Hartley, M.K., Hagan, C., Clark, N.L., and Elde, N.C. (2015). Overlapping Patterns of Rapid Evolution in the Nucleic Acid Sensors cGAS and OAS1 Suggest a Common Mechanism of Pathogen Antagonism and Escape. PLoS Genet. *11*, e1005203.
45. Mozzi, A., Pontremoli, C., Forni, D., Clerici, M., Pozzoli, U., Bresolin, N., Cagliani, R., and Sironi, M. (2015). OASes and STING: Adaptive Evolution in Concert. Genome Biol. Evol. *7*, 1016–1032.
46. Ding, Q., Gaska, J.M., Douam, F., Wei, L., Kim, D., Balev, M., Heller, B., and Ploss, A. (2018). Species-specific disruption of STING-dependent antiviral cellular defenses by the Zika virus NS2B3 protease. Proc. Natl. Acad. Sci. USA *115*, E6310–E6318.
47. Anggakusuma, Brown, R.J.P., Banda, D.H., Todt, D., Vieyres, G., Steinmann, E., and Pietschmann, T. (2016). Hepacivirus NS3/4A Proteases Interfere with MAVS Signaling in both Their Cognate Animal Hosts and Humans: Implications for Zoonotic Transmission. J. Virol. *90*, 10670–10681.
48. Stegelmeier, A.A., van Vloten, J.P., Mould, R.C., Klafuric, E.M., Minott, J.A., Wootton, S.K., Bridle, B.W., and Karimi, K. (2019). Myeloid Cells during Viral Infections and Inflammation. Viruses *11*, 168.
49. Cai, C., Tang, Y.-D., Xu, G., and Zheng, C. (2021). The crosstalk between viral RNA- and DNA-sensing mechanisms. Cell. Mol. Life Sci. *78*, 7427–7434.

50. Fan, Y.M., Zhang, Y.L., Luo, H., and Mohamud, Y. (2022). Crosstalk between RNA viruses and DNA sensors: Role of the cGAS-STING signalling pathway. Rev. Med. Virol. *32*, e2343.

51. Ni, G., Ma, Z., and Damania, B. (2018). cGAS and STING: At the intersection of DNA and RNA virus-sensing networks. PLoS Pathog. *14*, e1007148.

52. Neufeldt, C.J., Cerikan, B., Cortese, M., Frankish, J., Lee, J.-Y., Plociennikowska, A., Heigwer, F., Prasad, V., Joecks, S., Burkart, S.S., et al. (2022). SARS-CoV-2 infection induces a pro-inflammatory cytokine response through cGAS-STING and NF-κB. Commun. Biol. *5*, 45.

53. Ng, W.C., Kwek, S.S., Sun, B., Yousefi, M., Ong, E.Z., Tan, H.C., Puschnik, A.S., Chan, K.R., Ooi, Y.S., and Ooi, E.E. (2022). A fast-growing dengue virus mutant reveals a dual role of STING in response to infection. Open Biol. *12*, 220227.

54. Webb, L.G., and Fernandez-Sesma, A. (2022). RNA viruses and the cGAS-STING pathway: reframing our understanding of innate immune sensing. Curr. Opin. Virol. *53*, 101206.

55. Hertzog, J., Zhou, W., Fowler, G., Rigby, R.E., Bridgeman, A., Blest, H.T., Cursi, C., Chauveau, L., Davenne, T., Warner, B.E., et al. (2022). Varicella-Zoster virus ORF9 is an antagonist of the DNA sensor cGAS. EMBO J. *41*, e109217.

56. Zuniga, E.I., Macal, M., Lewis, G.M., and Harker, J.A. (2015). Innate and Adaptive Immune Regulation During Chronic Viral Infections. Annu. Rev. Virol. *2*, 573–597.

57. Baglio, S.R., van Eijndhoven, M.A.J., Koppers-Lalic, D., Berenguer, J., Lougheed, S.M., Gibbs, S., Léveillé, N., Rinkel, R.N.P.M., Hopmans, E.S., Swaminathan, S., et al. (2016). Sensing of latent EBV infection through exosomal transfer of 5′pppRNA. Proc. Natl. Acad. Sci. USA *113*, E587–E596.

58. Rousseau, B.A., and Bhaduri-McIntosh, S. (2023). Inflammation and Epstein–Barr Virus at the Crossroads of Multiple Sclerosis and Post-Acute Sequelae of COVID-19 Infection. Viruses *15*, 949.

59. Riou, R., Bressollette-Bodin, C., Boutoille, D., Gagne, K., Rodallec, A., Lefebvre, M., Raffi, F., Senitzer, D., Imbert-Marcille, B.-M., and Retière, C. (2017). Severe Symptomatic Primary Human Cytomegalovirus Infection despite Effective Innate and Adaptive Immune Responses. J. Virol. *91*, *022455*–e2316.

60. Dunmire, S.K., Odumade, O.A., Porter, J.L., Reyes-Genere, J., Schmeling, D.O., Bilgic, H., Fan, D., Baechler, E.C., Balfour, H.H., and Hogquist, K.A. (2014). Primary EBV Infection Induces an Expression Profile Distinct from Other Viruses but Similar to Hemophagocytic Syndromes. PLoS One *9*, e85422.

61. Fathi, N., and Rezaei, N. (2020). Lymphopenia in COVID-19: Therapeutic opportunities. Cell Biol. Int. *44*, 1792–1797.

62. Diao, B., Wang, C., Tan, Y., Chen, X., Liu, Y., Ning, L., Chen, L., Li, M., Liu, Y., Wang, G., et al. (2020). Reduction and Functional Exhaustion of T Cells in Patients With Coronavirus Disease 2019 (COVID-19). Front. Immunol. *11*, 827.

63. Kahan, S.M., Wherry, E.J., and Zajac, A.J. (2015). T cell exhaustion during persistent viral infections. Virology *479–480*, 180–193.

64. Iampietro, M., Younan, P., Nishida, A., Dutta, M., Lubaki, N.M., Santos, R.I., Koup, R.A., Katze, M.G., and Bukreyev, A. (2017). Ebola virus glycoprotein directly triggers T lymphocyte death despite of the lack of infection. PLoS Pathog. *13*, e1006397.

65. St John, A.L., and Rathore, A.P.S. (2019). Adaptive immune responses to primary and secondary dengue virus infections. Nat. Rev. Immunol. *19*, 218–230.

66. Tian, Y., Seumois, G., De-Oliveira-Pinto, L.M., Mateus, J., Herrera-de la Mata, S., Kim, C., Hinz, D., Goonawardhana, N.D.S., de Silva, A.D., Premawansa, S., et al. (2019). Molecular Signatures of Dengue Virus-Specific IL-10/IFN-γ Co-producing CD4 T Cells and Their Association with Dengue Disease. Cell Rep. *29*, 4482–4495.e4.

67. Grifoni, A., Voic, H., Yu, E.D., Mateus, J., Yan Fung, K.M., Wang, A., Seumois, G., De Silva, A.D., Tennekon, R., Premawansa, S., et al. (2022). Transcriptomics of Acute DENV-Specific CD8+ T Cells Does Not Support Qualitative Differences as Drivers of Disease Severity. Vaccines *10*, 612.

68. Tian, Y., Grifoni, A., Sette, A., and Weiskopf, D. (2019). Human T Cell Response to Dengue Virus Infection. Front. Immunol. *10*, 2125.

69. Hatch, S., Endy, T.P., Thomas, S., Mathew, A., Potts, J., Pazoles, P., Libraty, D.H., Gibbons, R., and Rothman, A.L. (2011). Intracellular Cytokine Production by Dengue Virus–specific T cells Correlates with Subclinical Secondary Infection. J. Infect. Dis. *203*, 1282–1291.

70. Screaton, G., Mongkolsapaya, J., Yacoub, S., and Roberts, C. (2015). New insights into the immunopathology and control of dengue virus infection. Nat. Rev. Immunol. *15*, 745–759.

71. Ratnasiri, K., Wilk, A.J., Lee, M.J., Khatri, P., and Blish, C.A. (2023). Single-cell RNA-seq methods to interrogate virus-host interactions. Semin. Immunopathol. *45*, 71–89.

72. Malhotra, S., Yen, J.Y., Honko, A.N., Garamszegi, S., Caballero, I.S., Johnson, J.C., Mucker, E.M., Trefry, J.C., Hensley, L.E., and Connor, J.H. (2013). Transcriptional profiling of the circulating immune response to lassa virus in an aerosol model of exposure. PLoS Neglected Trop. Dis. *7*, e2171.

73. Rasmussen, A.L., Tchitchek, N., Safronetz, D., Carter, V.S., Williams, C.M., Haddock, E., Korth, M.J., Feldmann, H., and Katze, M.G. (2015). Delayed Inflammatory and Cell Death Responses Are Associated with Reduced Pathogenicity in Lujo Virus-Infected Cynomolgus Macaques. J. Virol. *89*, 2543–2552.

74. Djavani, M.M., Crasta, O.R., Zapata, J.C., Fei, Z., Folkerts, O., Sobral, B., Swindells, M., Bryant, J., Davis, H., Pauza, C.D., et al. (2007). Early blood profiles of virus infection in a monkey model for Lassa fever. J. Virol. *81*, 7960–7973.

75. Connor, J.H., Yen, J., Caballero, I.S., Garamszegi, S., Malhotra, S., Lin, K., Hensley, L., and Goff, A.J. (2015). Transcriptional Profiling of the Immune Response to Marburg Virus Infection. J. Virol. *89*, 9865–9874.

76. Speranza, E., Bixler, S.L., Altamura, L.A., Arnold, C.E., Pratt, W.D., Taylor-Howell, C., Burrows, C., Aguilar, W., Rossi, F., Shamblin, J.D., et al. (2018). A conserved transcriptional response to intranasal Ebola virus exposure in nonhuman primates prior to onset of fever. Sci. Transl. Med. *10*, eaaq1016.

77. Yen, J.Y., Garamszegi, S., Geisbert, J.B., Rubins, K.H., Geisbert, T.W., Honko, A., Xia, Y., Connor, J.H., and Hensley, L.E. (2011). Therapeutics of Ebola Hemorrhagic Fever: Whole-Genome Transcriptional Analysis of Successful Disease Mitigation. J. Infect. Dis. *204*, S1043–S1052.

78. Versteeg, K., Menicucci, A.R., Woolsey, C., Mire, C.E., Geisbert, J.B., Cross, R.W., Agans, K.N., Jeske, D., Messaoudi, I., and Geisbert, T.W. (2017). Infection with the Makona variant results in a delayed and distinct host immune response compared to previous Ebola virus variants. Sci. Rep. *7*, 9730.

79. Speranza, E., Altamura, L.A., Kulcsar, K., Bixler, S.L., Rossi, C.A., Schoepp, R.J., Nagle, E., Aguilar, W., Douglas, C.E., Delp, K.L., et al. (2017). Comparison of Transcriptomic Platforms for Analysis of Whole Blood from Ebola-Infected Cynomolgus Macaques. Sci. Rep. *7*, 14756.

80. Broeckel, R.M., Feldmann, F., McNally, K.L., Chiramel, A.I., Sturdevant, G.L., Leung, J.M., Hanley, P.W., Lovaglio, J., Rosenke, R., Scott, D.P., et al. (2021). A pigtailed macaque model of Kyasanur Forest disease virus and Alkhurma hemorrhagic disease virus pathogenesis. PLoS Pathog. *17*, e1009678.

81. Aid, M., Abbink, P., Larocca, R.A., Boyd, M., Nityanandam, R., Nanayakkara, O., Martinot, A.J., Moseley, E.T., Blass, E., Borducchi, E.N., et al. (2017). Zika Virus Persistence in the Central Nervous System and Lymph Nodes of Rhesus Monkeys. Cell *169*, 610–620.e14.

82. Strouts, F.R., Popper, S.J., Partidos, C.D., Stinchcomb, D.T., Osorio, J.E., and Relman, D.A. (2016). Early Transcriptional Signatures of the

CellPress
OPEN ACCESS

Immune Response to a Live Attenuated Tetravalent Dengue Vaccine Candidate in Non-human Primates. PLoS Neglected Trop. Dis. *10*, e0004731.

83. de Wit, E., Rasmussen, A.L., Falzarano, D., Bushmaker, T., Feldmann, F., Brining, D.L., Fischer, E.R., Martellaro, C., Okumura, A., Chang, J., et al. (2013). Middle East respiratory syndrome coronavirus (MERS-CoV) causes transient lower respiratory tract infection in rhesus macaques. Proc. Natl. Acad. Sci. USA *110*, 16598–16603.
84. Aid, M., Busman-Sahay, K., Vidal, S.J., Maliga, Z., Bondoc, S., Starke, C., Terry, M., Jacobson, C.A., Wrijil, L., Ducat, S., et al. (2020). Vascular Disease and Thrombosis in SARS-CoV-2-Infected Rhesus Macaques. Cell *183*, 1354–1366.e13.
85. Coleman, C., Doyle-Meyers, L.A., Russell-Lodrigue, K.E., Golden, N., Threeton, B., Song, K., Pierre, G., Baribault, C., Bohm, R.P., Maness, N.J., et al. (2021). Similarities and Differences in the Acute-Phase Response to SARS-CoV-2 in Rhesus Macaques and African Green Monkeys. Front. Immunol. *12*, 754642.
86. Liu, X., Speranza, E., Muñoz-Fontela, C., Haldenby, S., Rickett, N.Y., Garcia-Dorival, I., Fang, Y., Hall, Y., Zekeng, E.-G., Lüdtke, A., et al. (2017). Transcriptomic signatures differentiate survival from fatal outcomes in humans infected with Ebola virus. Genome Biol. *18*, 4.
87. Soares-Schanoski, A., Baptista Cruz, N., de Castro-Jorge, L.A., de Carvalho, R.V.H., de, Santos, C.A., dos, Rós, N., da, Oliveira, Rós, N.d., Oliveira, Ú., Costa, D.D., Santos, C.L.S.D., Cunha, M.D.P., et al. (2019). Systems analysis of subjects acutely infected with the Chikungunya virus. PLoS Pathog. *15*, e1007880.
88. Michlmayr, D., Pak, T.R., Rahman, A.H., Amir, E.A.D., Kim, E.Y., Kim-Schulze, S., Suprun, M., Stewart, M.G., Thomas, G.P., Balmaseda, A., et al. (2018). Comprehensive innate immune profiling of chikungunya virus infection in pediatric cases. Mol. Syst. Biol. *14*, e7862.
89. Thair, S.A., He, Y.D., Hasin-Brumshtein, Y., Sakaram, S., Pandya, R., Toh, J., Rawling, D., Remmel, M., Coyle, S., Dalekos, G.N., et al. (2021). Transcriptomic similarities and differences in host response between SARS-CoV-2 and other viral infections. iScience *24*, 101947.
90. de Steenhuijsen Piters, W.A.A., Heinonen, S., Hasrat, R., Bunsow, E., Smith, B., Suarez-Arrabal, M.-C., Chaussabel, D., Cohen, D.M., Sanders, E.A.M., Ramilo, O., et al. (2016). Nasopharyngeal Microbiota, Host Transcriptome, and Disease Severity in Children with Respiratory Syncytial Virus Infection. Am. J. Respir. Crit. Care Med. *194*, 1104–1115.
91. Liu, T.-Y., Burke, T., Park, L.P., Woods, C.W., Zaas, A.K., Ginsburg, G.S., and Hero, A.O. (2016). An individualized predictor of health and disease using paired reference and target samples. BMC Bioinf. *17*, 47.
92. Zhai, Y., Franco, L.M., Atmar, R.L., Quarles, J.M., Arden, N., Bucasas, K.L., Wells, J.M., Niño, D., Wang, X., Zapata, G.E., et al. (2015). Host Transcriptional Response to Influenza and Other Acute Respiratory Viral Infections – A Prospective Cohort Study. PLoS Pathog. *11*, e1004869.
93. Jaggi, P., Mejias, A., Xu, Z., Yin, H., Moore-Clingenpeel, M., Smith, B., Burns, J.C., Tremoulet, A.H., Jordan-Villegas, A., Chaussabel, D., et al. (2018). Whole blood transcriptional profiles as a prognostic tool in complete and incomplete Kawasaki Disease. PLoS One *13*, e0197858.
94. Heinonen, S., Jartti, T., Garcia, C., Oliva, S., Smitherman, C., Anguiano, E., de Steenhuijsen Piters, W.A.A., Vuorinen, T., Ruuskanen, O., Dimo, B., et al. (2016). Rhinovirus Detection in Symptomatic and Asymptomatic Children: Value of Host Transcriptome Analysis. Am. J. Respir. Crit. Care Med. *193*, 772–782.
95. Sweeney, T.E., Shidham, A., Wong, H.R., and Khatri, P. (2015). A comprehensive time-course–based multicohort analysis of sepsis and sterileinflammation reveals a robust diagnostic gene set. Sci. Transl. Med. *7*, 287ra71.
96. Wong, H.R., Shanley, T.P., Sakthivel, B., Cvijanovich, N., Lin, R., Allen, G.L., Thomas, N.J., Doctor, A., Kalyanaraman, M., Tofil, N.M., et al. (2007). Genome-level expression profiles in pediatric septic shock indicate a role for altered zinc homeostasis in poor outcome. Physiol. Genom. *30*, 146–155.
97. Wong, H.R., Cvijanovich, N.Z., Hall, M., Allen, G.L., Thomas, N.J., Freishtat, R.J., Anas, N., Meyer, K., Checchia, P.A., Lin, R., et al. (2012). Interleukin-27 is a novel candidate diagnostic biomarker for bacterial infection in critically ill children. Crit. Care *16*, R213.
98. Wong, H.R., Cvijanovich, N., Wheeler, D.S., Bigham, M.T., Monaco, M., Odoms, K., Macias, W.L., and Williams, M.D. (2008). Interleukin-8 as a Stratification Tool for Interventional Trials Involving Pediatric Septic Shock. Am. J. Respir. Crit. Care Med. *178*, 276–282.
99. Ramilo, O., Allman, W., Chung, W., Mejias, A., Ardura, M., Glaser, C., Wittkowski, K.M., Piqueras, B., Banchereau, J., Palucka, A.K., and Chaussabel, D. (2007). Gene expression patterns in blood leukocytes discriminate patients with acute infections. Blood *109*, 2066–2077.
100. Hoang, L.T., Tolfvenstam, T., Ooi, E.E., Khor, C.C., Naim, A.N.M., Ho, E.X.P., Ong, S.H., Wertheim, H.F., Fox, A., Van Vinh Nguyen, C., et al. (2014). Patient-Based Transcriptome-Wide Analysis Identify Interferon and Ubiquination Pathways as Potential Predictors of Influenza A Disease Severity. PLoS One *9*, e111640.
101. Davenport, E.E., Antrobus, R.D., Lillie, P.J., Gilbert, S., and Knight, J.C. (2015). Transcriptomic profiling facilitates classification of response to influenza challenge. J. Mol. Med. *93*, 105–114.
102. Parnell, G.P., McLean, A.S., Booth, D.R., Armstrong, N.J., Nalos, M., Huang, S.J., Manak, J., Tang, W., Tam, O.-Y., Chan, S., and Tang, B.M. (2012). A distinct influenza infection signature in the blood transcriptome of patients with severe community-acquired pneumonia. Crit. Care *16*, R157.
103. Mejias, A., Dimo, B., Suarez, N.M., Garcia, C., Suarez-Arrabal, M.C., Jartti, T., Blankenship, D., Jordan-Villegas, A., Ardura, M.I., Xu, Z., et al. (2013). Whole Blood Gene Expression Profiles to Assess Pathogenesis and Disease Severity in Infants with Respiratory Syncytial Virus Infection. PLoS Med. *10*, e1001549.
104. Wang, Y., Dennehy, P.H., Keyserling, H.L., Tang, K., Gentsch, J.R., Glass, R.I., and Jiang, B. (2007). Rotavirus Infection Alters Peripheral T-Cell Homeostasis in Children with Acute Diarrhea. J. Virol. *81*, 3904–3912.
105. Berdal, J.-E., Mollnes, T.E., Wæhre, T., Olstad, O.K., Halvorsen, B., Ueland, T., Laake, J.H., Furuseth, M.T., Maagaard, A., Kjekshus, H., et al. (2011). Excessive innate immune response and mutant D222G/N in severe A (H1N1) pandemic influenza. J. Infect. *63*, 308–316.
106. Dickinson, P., Smith, C.L., Forster, T., Craigon, M., Ross, A.J., Khondoker, M.R., Ivens, A., Lynn, D.J., Orme, J., Jackson, A., et al. (2015). Whole blood gene expression profiling of neonates with confirmed bacterial sepsis. Genom. Data *3*, 41–48.
107. Bermejo-Martin, J.F., Martin-Loeches, I., Rello, J., Antón, A., Almansa, R., Xu, L., Lopez-Campos, G., Pumarola, T., Ran, L., Ramirez, P., et al. (2010). Host adaptive immunity deficiency in severe pandemic influenza. Crit. Care *14*, R167.
108. Parnell, G., McLean, A., Booth, D., Huang, S., Nalos, M., and Tang, B. (2011). Aberrant Cell Cycle and Apoptotic Changes Characterise Severe Influenza A Infection – A Meta-Analysis of Genomic Signatures in Circulating Leukocytes. PLoS One *6*, e17186.
109. Zaas, A.K., Chen, M., Varkey, J., Veldman, T., Hero, A.O., Lucas, J., Huang, Y., Turner, R., Gilbert, A., Lambkin-Williams, R., et al. (2009). Gene Expression Signatures Diagnose Influenza and Other Symptomatic Respiratory Viral Infections in Humans. Cell Host Microbe *6*, 207–217.
110. Yu, J., Peterson, D.R., Baran, A.M., Bhattacharya, S., Wylie, T.N., Falsey, A.R., Mariani, T.J., and Storch, G.A. (2019). Host Gene Expression in Nose and Blood for the Diagnosis of Viral Respiratory Infection. J. Infect. Dis. *219*, 1151–1161.
111. Dunning, J., Blankley, S., Hoang, L.T., Cox, M., Graham, C.M., James, P.L., Bloom, C.I., Chaussabel, D., Banchereau, J., Brett, S.J., et al. (2018). Progression of whole-blood transcriptional signatures from interferon-induced to neutrophil-associated patterns in severe influenza. Nat. Immunol. *19*, 625–635.

112. Rodriguez-Fernandez, R., Tapia, L.I., Yang, C.-F., Torres, J.P., Chavez-Bueno, S., Garcia, C., Jaramillo, L.M., Moore-Clingenpeel, M., Jafri, H.S., Peeples, M.E., et al. (2017). Respiratory Syncytial Virus Genotypes, Host Immune Profiles, and Disease Severity in Young Children Hospitalized With Bronchiolitis. J. Infect. Dis. *217*, 24–34.

113. Tang, B.M., Shojaei, M., Teoh, S., Meyers, A., Ho, J., Ball, T.B., Keynan, Y., Pisipati, A., Kumar, A., Eisen, D.P., et al. (2019). Neutrophils-related host factors associated with severe disease and fatality in patients with influenza infection. Nat. Commun. *10*, 3422.

114. Jong, V.L., Ahout, I.M.L., van den Ham, H.J., Jans, J., Zaaraoui-Boutahar, F., Zomer, A., Simonetti, E., Bijl, M.A., Brand, H.K., van IJcken, W.F.J., et al. (2016). Transcriptome assists prognosis of disease severity in respiratory syncytial virus infected infants. Sci. Rep. *6*, 36603.

115. Simmons, C.P., Popper, S., Dolocek, C., Chau, T.N.B., Griffiths, M., Dung, N.T.P., Long, T.H., Hoang, D.M., Chau, N.V., Thao, L.T.T., et al. (2007). Patterns of Host Genome—Wide Gene Transcript Abundance in the Peripheral Blood of Patients with Acute Dengue Hemorrhagic Fever. J. Infect. Dis. *195*, 1097–1107.

116. Long, H.T., Hibberd, M.L., Hien, T.T., Dung, N.M., Van Ngoc, T., Farrar, J., Wills, B., and Simmons, C.P. (2009). Patterns of gene transcript abundance in the blood of children with severe or uncomplicated dengue highlight differences in disease evolution and host response to dengue virus infection. J. Infect. Dis. *199*, 537–546.

117. Popper, S.J., Gordon, A., Liu, M., Balmaseda, A., Harris, E., and Relman, D.A. (2012). Temporal Dynamics of the Transcriptional Response to Dengue Virus Infection in Nicaraguan Children. PLoS Neglected Trop. Dis. *6*, e1966.

118. Kwissa, M., Nakaya, H.I., Onlamoon, N., Wrammert, J., Villinger, F., Perng, G.C., Yoksan, S., Pattanapanyasat, K., Chokephaibulkit, K., Ahmed, R., and Pulendran, B. (2014). Dengue Virus Infection Induces Expansion of a CD14+CD16+ Monocyte Population that Stimulates Plasmablast Differentiation. Cell Host Microbe *16*, 115–127.

119. DeBerg, H.A., Zaidi, M.B., Altman, M.C., Khaenam, P., Gersuk, V.H., Campos, F.D., Perez-Martinez, I., Meza-Segura, M., Chaussabel, D., Banchereau, J., et al. (2018). Shared and organism-specific host responses to childhood diarrheal diseases revealed by whole blood transcript profiling. PLoS One *13*, e0192082.

120. Sellers, S.A., Fischer, W.A., Heise, M.T., and Schughart, K. (2021). Highly dampened blood transcriptome response in HIV patients after respiratory infection. Sci. Rep. *11*, 4465.

121. Bolen, C.R., Robek, M.D., Brodsky, L., Schulz, V., Lim, J.K., Taylor, M.W., and Kleinstein, S.H. (2013). The Blood Transcriptional Signature of Chronic Hepatitis C Virus Is Consistent with an Ongoing Interferon-Mediated Antiviral Response. J. Interferon Cytokine Res. *33*, 15–23.

122. Satija, R., Farrell, J.A., Gennert, D., Schier, A.F., and Regev, A. (2015). Spatial reconstruction of single-cell gene expression data. Nat. Biotechnol. *33*, 495–502.

123. Love, M.I., Huber, W., and Anders, S. (2014). Moderated estimation of fold change and dispersion for RNA-seq data with DESeq2. Genome Biol. *15*, 550.

124. Gu, Z. (2022). Complex heatmap visualization. iMeta.

125. Kuznetsova, A., Brockhoff, P.B., and Christensen, R.H.B. (2017). lmerTest Package: Tests in Linear Mixed Effects Models. J. Stat. Software *82*.

126. Wolf, F.A., Angerer, P., and Theis, F.J. (2018). SCANPY: large-scale single-cell gene expression data analysis. Genome Biol. *19*, 15.

127. Wickham, H. (2011). ggplot2. WIREs Computational Stats. *3*, 180–185.

128. McInnes, L., Healy, J., and Melville, J. (2018). UMAP: Uniform Manifold Approximation and Projection for Dimension Reduction. Preprint at arXiv. https://doi.org/10.48550/arxiv.1802.03426.

129. Soneson, C., Love, M.I., and Robinson, M.D. (2015). Differential analyses for RNA-seq: transcript-level estimates improve gene-level inferences. F1000Res. *4*, 1521.

130. Patro, R., Duggal, G., Love, M.I., Irizarry, R.A., and Kingsford, C. (2017). Salmon provides fast and bias-aware quantification of transcript expression. Nat. Methods *14*, 417–419.

131. R Core Team (2021). R: A Language and Environment for Statistical ## Computing (R Foundation for Statistical Computing). https://www.R-project.org/.

132. Buturovic, L., Zheng, H., Tang, B., Lai, K., Kuan, W.S., Gillett, M., Santram, R., Shojaei, M., Almansa, R., Nieto, J.Á., et al. (2022). A 6-mRNA host response classifier in whole blood predicts outcomes in COVID-19 and other acute viral infections. Sci. Rep. *12*, 889.

133. Liu, Y.E., Saul, S., Rao, A.M., Robinson, M.L., Agudelo Rojas, O.L., Sanz, A.M., Verghese, M., Solis, D., Sibai, M., Huang, C.H., et al. (2022). An 8-gene machine learning model improves clinical prediction of severe dengue progression. Genome Med. *14*, 33.

134. Sweeney, T.E., Wong, H.R., and Khatri, P. (2016). Robust classification of bacterial and viral infections via integrated host gene expression diagnostics. Sci. Transl. Med. *8*, 346ra91.

135. Ali, A., Rasheed, A., Siddiqui, A.A., Naseer, M., Wasim, S., and Akhtar, W. (2015). Non-Parametric Test for Ordered Medians: The Jonckheere Terpstra Test. Int. J. Stat. Med. Res. *4*, 203–207.

136. Hagan, T., Gerritsen, B., Tomalin, L.E., Fourati, S., Mulè, M.P., Chawla, D.G., Rychkov, D., Henrich, E., Miller, H.E.R., Diray-Arce, J., et al. (2022). Transcriptional atlas of the human immune response to 13 vaccines reveals a common predictor of vaccine-induced antibody responses. Nat. Immunol. *23*, 1788–1798.

137. Ghita, L., Yao, Z., Xie, Y., Duran, V., Cagirici, H.B., Samir, J., Osman, I., Rojas, O.L.A., Sanz, A.M., Sahoo, M.K., et al. (2023). Global and cell type-specific immunological hallmarks of severe dengue progression. Preprint at bioRxiv. https://doi.org/10.1101/2022.12.11.519930.

138. Hao, Y., Hao, S., Andersen-Nissen, E., Mauck, W.M., Zheng, S., Butler, A., Lee, M.J., Wilk, A.J., Darby, C., Zager, M., et al. (2021). Integrated analysis of multimodal single-cell data. Cell *184*, 3573–3587.e29.

STAR★METHODS

KEY RESOURCES TABLE

REAGENT or RESOURCE	SOURCE	IDENTIFIER
Deposited data		
Microarray dataset	Fukuyama et al.[14]	accession# GSE152406
Microarray dataset	Skinner et al.[15]	accession# GSE60009
Microarray dataset	Malhotra et al.[72]	accession# GSE41752
Microarray dataset	Rasmussen et al.[73]	accession# GSE49838
Microarray dataset	Djavani et al.[74]	accession# GSE5790
Microarray dataset	Connor et al.[75]	accession# GSE58287
RNA-seq dataset	Speranza et al.[76]	accession# GSE103825
Microarray dataset	Yen et al.[77]	accession# GSE24943
RNA-seq dataset	Maroney et al.[13]	accession# PRJNA718880
RNA-seq dataset	Versteeg et al.[78]	accession# PRJNA398558
RNA-seq dataset	Speranza et al.[79]	accession# GSE99463
RNA-seq dataset	Broeckel et al.[80]	accession# GSE185797
Microarray dataset	Aid et al.[81]	accession# GSE90868
Microarray dataset	Strouts et al.[82]	accession# GSE72430
Microarray dataset	de Wit et al.[83]	accession# GSE44542
RNA-seq dataset	Aid et al.[84]	accession# GSE156701
RNA-seq dataset	Price et al.	accession# GSE155363
RNA-seq dataset	Coleman et al.[85]	accession# GSE184949_GPL29319
RNA-seq dataset	Reynard et al.[11]	Zenodo# 7229439
scRNA-seq dataset	Kotliar et al.[16]	accession# GSE158390
scRNA-seq dataset	Ghita et al.[23]	accession# GSE220969
scRNA-seq dataset	Yoshida et al.[22]	accession# EGAD00001007718
RNA-seq dataset	Liu et al.[86]	accession# PRJNA352396
RNA-seq dataset	Soares-Schanoski et al.[87]	accession# PRJNA507472
RNA-seq dataset	Michlmayr et al.[88]	accession# PRJNA390289
RNA-seq dataset	Thair et al.[89]	accession# GSE152641
Microarray dataset	de Steenhuijsen Piters et al.[90]	accession# GSE77087
Microarray dataset	Liu et al.[91]	accession# GSE73072
Microarray dataset	Zhai et al.[92]	accession# GSE68310
Microarray dataset	Jaggi et al.[93]	accession# GSE68004
Microarray dataset	Heinonen et al.[94]	accession# GSE67059
Microarray dataset	Sweeney et al.[95]	accession# GSE66099
Microarray dataset	Wong et al., Wong et al., Wong et al.[96–98]	accession# GSE4607
Microarray dataset	Ramilo et al.[99]	accession# GSE6269
Microarray dataset	Hoang et al.[100]	accession# GSE61821
Microarray dataset	Davenport et al.[101]	accession# GSE61754
Microarray dataset	Parnell et al.[102]	accession# GSE40012
Microarray dataset	Mejias et al.[103]	accession# GSE38900
Microarray dataset	Wang et al.[104]	accession# GSE2729
Microarray dataset	Berdal et al.[105]	accession# GSE27131
Microarray dataset	Dickinson[106]	accession# GSE25504
Microarray dataset	Bermejo-Martin et al.[107]	accession# GSE21802
Microarray dataset	Parnell et al.[108]	accession# GSE20346
Microarray dataset	Zaas et al.[109]	accession# GSE17156

(*Continued on next page*)

Continued

REAGENT or RESOURCE	SOURCE	IDENTIFIER
Microarray dataset	Yu et al.[110]	accession# GSE117827
Microarray dataset	Dunning et al.[111]	accession# GSE111368
Microarray dataset	Rodriguez-Fernandez et al.[112]	accession# GSE103842
Microarray dataset	Tang et al.[113]	accession# GSE101702
Microarray dataset	Jong et al.[114]	accession# E-MTAB-5195
Microarray dataset	Simmons et al.[115]	accession# GSE40628
Microarray dataset	Long et al.[116]	accession# GSE13052
Microarray dataset	Popper et al.[117]	accession# GSE38246
Microarray dataset	Kwissa et al.[118]	accession# GSE51808
RNA-seq dataset	DaBerg et al.[119]	accession# GSE69529
RNA-seq dataset	Sellers et al.[120]	accession# GSE155352
Microarray dataset	N/A	accession# GSE58208
Microarray dataset	Dunmire et al.[60]	accession# GSE45924_GLP6883
Microarray dataset	Riou et al.[59]	accession# GSE81246
Microarray dataset	Bolen et al.[121]	accession# GSE40224
**Software and algorithms**		
MetaIntegrator	Haynes et al.[21]	https://cran.r-project.org/web/packages/MetaIntegrator/index.html
Seurat	Satija et al.[122]	https://satijalab.org/seurat/; RRID:SCR_007322
DESeq2	Love et al.[123]	https://bioconductor.org/packages/release/bioc/html/DESeq2.html; RRID:SCR_015687
ComplexHeatmap	Gu[124]	https://bioconductor.org/packages/release/bioc/html/ComplexHeatmap.html; RRID:SCR_017270
lmerTest	Kuznetsova et al.[125]	https://cran.r-project.org/web/packages/lmerTest/index.html; RRID:SCR_015656
scanpy	Wolf et al.[126]	https://scanpy.readthedocs.io/en/stable/; RRID:SCR_018139
ggplot2	Wickham[127]	https://ggplot2.tidyverse.org; RRID:SCR_014601
UMAP	McInnes et al.[128]	https://cran.r-project.org/web/packages/umap/index.html; RRID:SCR_018217
Tximport	Soneson[129]	https://bioconductor.org/packages/release/bioc/html/tximport.html; RRID:SCR_016752
Salmon	Patro et al.[130]	https://combine-lab.github.io/salmon/; RRID:SCR_017036
R	R Core Team[131]	https://www.r-project.org/; RRID:SCR_001905

RESOURCE AVAILABILITY

Lead contact

Further information and requests for resources, software, and data should be directed to and will be fulfilled by the Lead Contact, Purvesh Khatri (pkhatri@stanford.edu).

Materials availability

This study did not generate new unique reagents.

Data and code availability

This study did not generate any unique datasets or code. All datasets, software, and algorithms used in this study are publicly available and listed in the Key Resource table. Code used to generate figures is available on Github: https://github.com/Khatri-Lab/NHP_virus_challenge and on Zenodo: https://doi.org/10.5281/zenodo.10420934. Any additional information required to reanalyze the data reported in this paper is available from the lead contact upon request.

METHOD DETAILS

Methods for analyses performed are described below.

QUANTIFICATION AND STATISTICAL ANALYSIS

Non-human primate dataset collection and preprocessing

We downloaded 21 gene expression datasets (either microarray or RNA-seq) from the National Center for Biotechnology Information (NCBI) Gene Expression Omnibus (GEO), Sequence Read Archive (SRA) or shared by collaborators, consisting of 743 samples derived from whole blood or peripheral blood mononuclear cells (PBMCs) (Tables 1 and S1). The counts dataset generated by Reynard et al.[11] was downloaded from Zenodo. The samples in these datasets included all available macaque virus challenge studies with samples from uninfected and two or more infected timepoints. We incorporated technical heterogeneity in our analysis as these datasets were profiled using microarray and RNA sequencing (RNA-seq) from different manufacturers. We did not renormalize custom arrays and used preprocessed data when made publicly available by the study authors. For microarray datasets, if a probe matched more than one gene, we expanded the expression data for that probe to add one record for each gene. When multiple probes mapped to the same gene within a dataset, we applied a fixed-effect model. Within a dataset, cohorts assayed with different microarray types were treated as independent.

If only raw RNA-seq sequencing files were available, reads were trimmed of Illumina adaptors and reads that were too short after adaptor trimming (less than 20 nt) were removed using Trim Galore (v0.6.5). We then mapped the cleaned reads to the macaque transcriptome (Salmon v1.3.0, genome version Mmul_10 or Macaca_fascicularis_6.0).[130] We used Tximport (v1.26.1)[129] to summarize to gene-level expression. We applied the variance stabilizing transformation from DESeq2 (v1.38.2)[123] to normalize gene expression of RNA-seq raw count data for downstream analysis and visualization.

We mapped all genes generated through alignment to macaque genomes (rhesus: Mmul_10, cynomolgus: Macaca_fascicularis_6.0, pig-tailed: Mnem_1.0) to the corresponding human orthologs to facilitate integrated, comparative analyses. Within a dataset, cohorts assayed with different microarray types or had different viruses with independent control animals were treated as independent. For the scRNA-seq data, authors of Kotliar et al.[16] generously shared their scRNA-seq object of GSE158390 that was processed and annotated for cell type.

Human dataset collection and preprocessing

We utilized human gene expression data collected and processed from our other studies.[8,132,133] This included 47 gene expression datasets from the National Center for Biotechnology Information (NCBI) Gene Expression Omnibus (GEO), Sequence Read Archive (SRA), ArrayExpress, and European Nucleotide Archive (ENA), consisting of 5,345 samples derived from whole blood or peripheral blood mononuclear cells (PBMCs) (Table S3). Log2 transformation and quantile normalization was applied when necessary. For the combined dataset from Zheng et al.[8] (Table S3) and separately the human dengue datasets,[115–118] Combat CONormalization Using conTrols (COCONUT) was used for between-dataset normalization.[134] Healthy samples from each cohort undergo ComBat co-normalization without covariates, and the ComBat estimated parameters are computed for the healthy samples in each dataset. By applying these parameters to the non-healthy samples, all datasets keep the same background distribution while retaining the same relative distance between healthy and disease samples, which preserves the biological variability between the two groups within a dataset. All other datasets were used individually and not co-normalized to reduce loss of genes.

Comparison of baseline macaque gene expression

To understand whether macaque species were comparable across gene expression, we compared differences in gene expression patterns across datasets containing the same macaque species to datasets containing a different macaque species. To do this, we utilized the union of genes across all datasets (3,055 shared genes). For each dataset, the mean and median of each gene was calculated. Pairwise spearman correlations were calculated across each dataset and the spearman correlation coefficients were plotted by macaque species comparison performed. Shapiro-Wilk test for normality was performed and Kruskal–Wallis test was performed across macaque species comparison groups. Pairwise wilcoxon tests were also performed with Bonferroni correction.

Gene signatures and scoring

We used a number of previously published gene signatures, including: MVS,[7] MS1 signature,[10] T cell activation signature,[19] and ISG signature.[18] Genes used for each signature are also included as Table S2. We generated gene signatures across all macaque datasets, the RNA virus subset of the datasets, and each viral family subset of the datasets using the MetaIntegrator workflow. Briefly,

score generation was done by applying two meta-analysis methods previously described: (1) combining effect sizes and (2) combining p values. To generate robust, comparable gene signatures per virus, we filtered signatures for effect size of 0.6 and false discovery rate (FDR) thresholds between 0.05 and 0.0001 in order to find thresholds that captured around 200 genes for better comparison across gene signatures. For the Viral Response Signature (VRS) that was generated across all macaque datasets, we removed one dataset at a time and applied both meta-analysis methods at each iteration to avoid the influence of any datasets with large sample sizes on the results.

We defined each signature score by the geometric mean of the normalized, log2-transformed expression of the overexpressed genes minus the geometric mean of the normalized, log2-transformed expression of the underexpressed genes of each gene signature. We scaled and centered (mean = 1, standard deviation = 1) all sample scores per dataset to allow for comparison between datasets.

We measured the correlation between different scores using Spearman's rank correlation coefficient. We used the Mann–Whitney U test (Wilcoxon rank-sum test) to compare MVS scores between two groups.

Jonckheere-Terpstra (JT) trend test[135] was used to assess the significance of the trend of scores over severity.

Mixed-effects model for time point data

We used multivariable linear mixed-effects models with random time-influenced subject-specific intercepts and slopes to assess the changes in MVS scores from uninfected baseline timepoints (intercepts), and follow-up timepoints post-infection (slopes). Separate models for were estimated using time, day-post-infection * day-post-infection, macaque species, dataset, and infecting viruses that included various interactions between these covariates. Across these various models, the one we chose was that with the lowest Akaike Information Criterion (AIC) value. The final reported model for both the macaque and the human datasets was: lmer formula = MVS_score $\sim$ Time + Time^2 + Virus + Time*Virus_Family + Time^2*Virus_Family+(1+Time|Subject). Analyses were run in R version 4.2.2 using the "lmerTest" package.

Gene set overrepresentation enrichment analysis

Overrepresentation analysis was performed on differentially expressed gene sets or signature sets identified from bulk RNA-seq (padj <0.05 and ES $\geq$ 0.1) and/or scRNA-seq (padj <0.05 and ES $\geq$ 0.6) analyses utilizing the Blood Transcriptional Modules (BTM).[19] Overrepresentation analysis was performed on upregulated or overrepresented genes separately from the downregulated or underrepresented genes. BTMs for which there was higher level annotation from Hagan et al.[136] were visualized in Circos plots and single cell analysis. The p values were adjusted using Bonferroni correction. BTM scores for Figure 3 were generated using the effect sizes from the meta-analysis performed on each virus family at peak timepoints as defined by DEG number.

Analysis of single-cell RNA sequencing

Data from Kotliar et al.[16] was generously shared as a processed object with cell types already assigned. We generated gene scores per cell type utilizing the geometric mean of the genes in the signature. Processed data from Yoshida et al.[22] was downloaded from GEO. Data from Ghita et al.[137] was generously shared. Both datasets were processed via Seurat and scanpy for QC, dimension reduction, clustering, and cell type classification. Seurat v4[138] was used for cell type annotation utilizing the multimodal PBMC reference dataset from the associated publication, and cell type calls were compared to previous manual annotation of datasets for confirmation. We generated MVS[7] and Module 4[8] scores per celltype across both datasets using the geometric mean of the genes in the subset. We utilized the FindMarkers function in Seurat to perform DEG analysis on each viral infected individual compared with all healthy controls in each dataset by cell type. We then performed BTM enrichment analyses per individual on the gene subset that was upregulated upon infection and the gene subset that was downregulated upon infection separately (padj <0.05 and ES $\geq$ 0.6). The pathway direction that had the highest adjusted pvalue was retained if it appeared in both the up and down regulated module list.

Figure generation

Figures were generated in R using the "ggplot2" and "ComplexHeatmap" package. Colors for figures were generated using the "NatParksPalettes"package. Statistical analyses were performed as described in figure and table legends and plotted using the R "ggpubr' package.

Cell Reports, Volume *43*

Supplemental information

Systems immunology of transcriptional responses to viral infection identifies conserved antiviral pathways across macaques and humans

Kalani Ratnasiri, Hong Zheng, Jiaying Toh, Zhiyuan Yao, Veronica Duran, Michele Donato, Mario Roederer, Megha Kamath, John-Paul M. Todd, Matthew Gagne, Kathryn E. Foulds, Joseph R. Francica, Kizzmekia S. Corbett, Daniel C. Douek, Robert A. Seder, Shirit Einav, Catherine A. Blish, and Purvesh Khatri

SUPPLEMENTAL FIGURES and TABLES

SFig1: Gene pair correlation comparison across the macaques

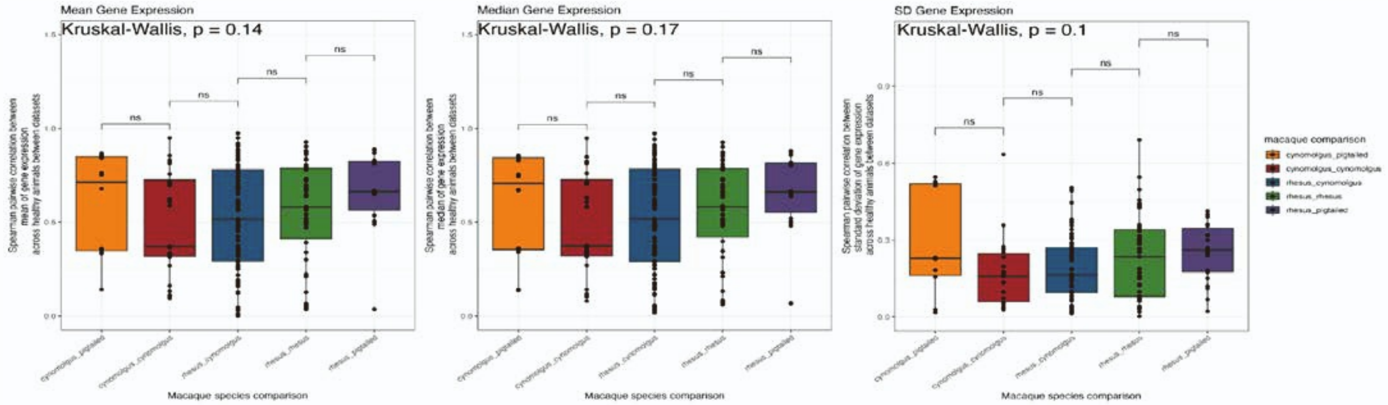

Correlation coefficients between the (left) mean (center) median and (right) standard deviation of gene expression of healthy animals per dataset in comparison to other datasets from the same or other macaque species. Pairwise wilcoxon test with bonferroni correction represented.

SFig2. AUROCs by dataset

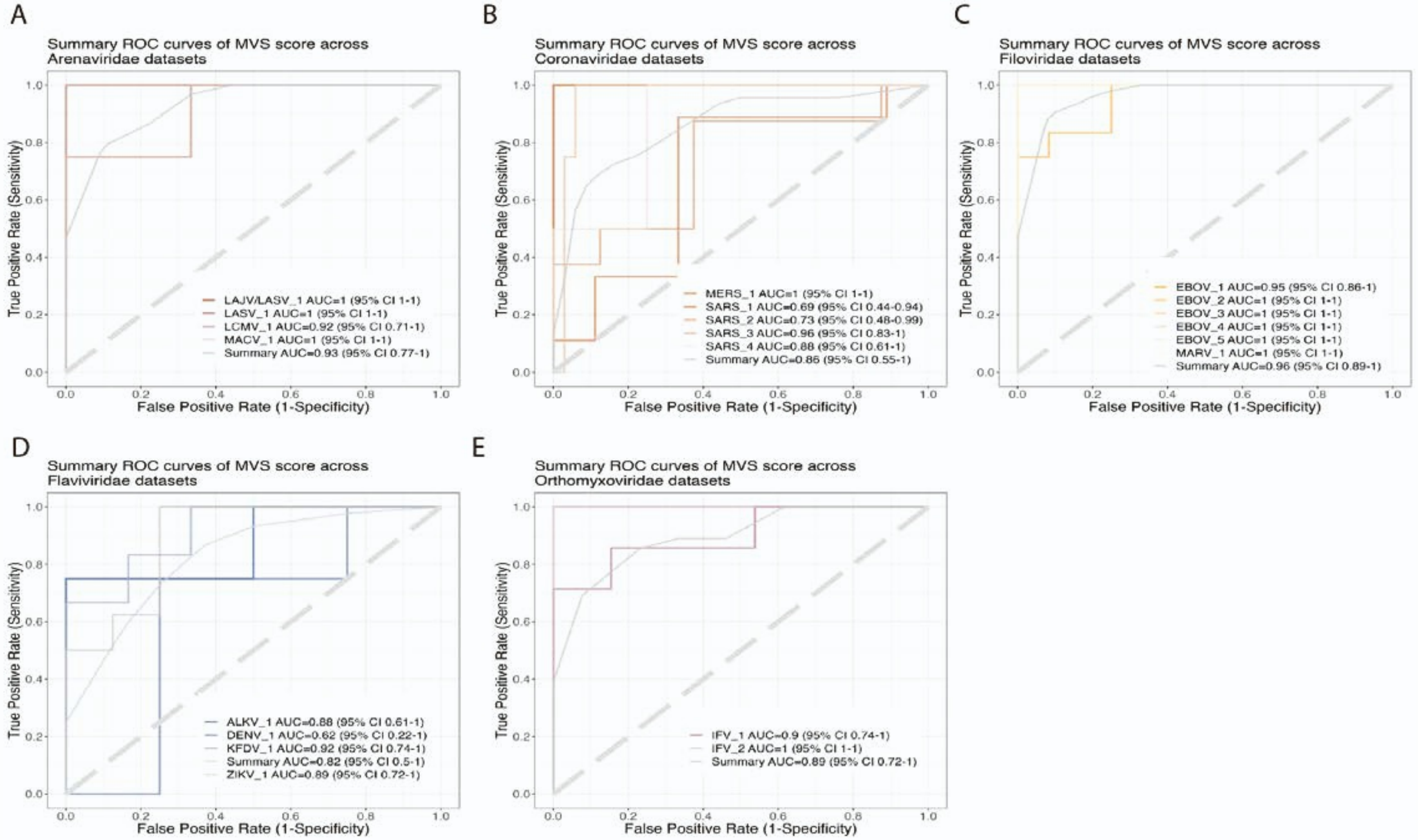

(A-E) ROC curves for distinguishing macaques with viral infection at peak MVS timepoint category from uninfected macaques, across datasets by viral family and colored by individual dataset.

SFig3. Validation of independent inflammation and monocyte and T cell function scores in NHP data

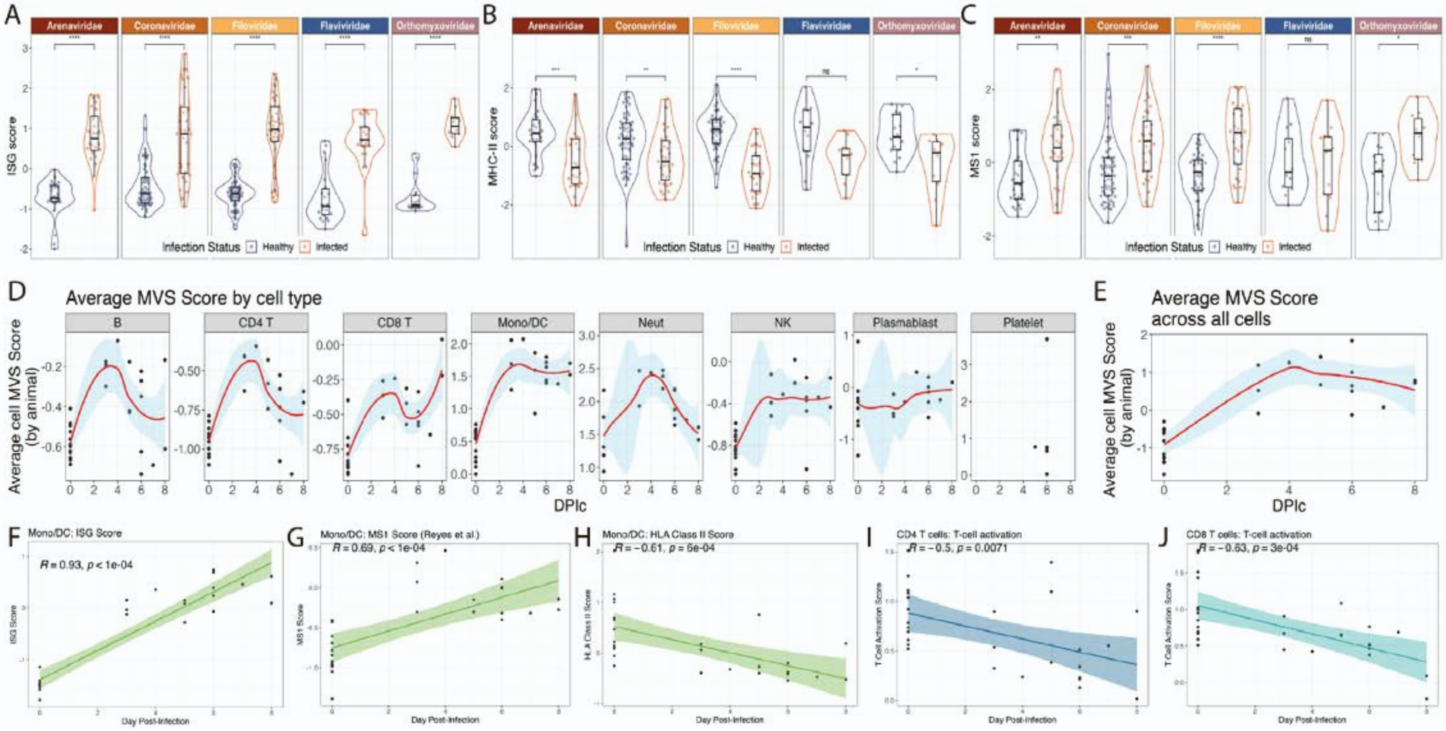

(A-J) Gene score by sample taking the geometric mean of the genes present and scaled across samples. **(A-C)** Distribution of the **(A)** ISG score, **(B)** HLA Class II Score and **(C)** MS1 Score comparing uninfected, healthy macaques to those at peak MVS score by viruses across five viral families. Each point represents a blood sample. Significance values were determined using an unpaired, one-sided Wilcoxon ranked-sum test with Bonferroni correction for multiple hypothesis testing. (**D–E)** Average MVS score by **(D)** cell type and **(E)** across all cells of the scRNA-seq EBOV infection NHP data from Figure 1H-K. **(F-J)** Correlation of average (**F)** ISG, **(G)** HLA class II and **(H)** MS1 scores in the Monocyte/DC population from Figure 1H with time post-infection. **(G-H)** Correlation of average T cell activation score in the **(I)** CD4 T cells and **(J)** CD8 T cell populations from Figure 1H with time post-infection.

SFig4. MVS score across all data timepoints

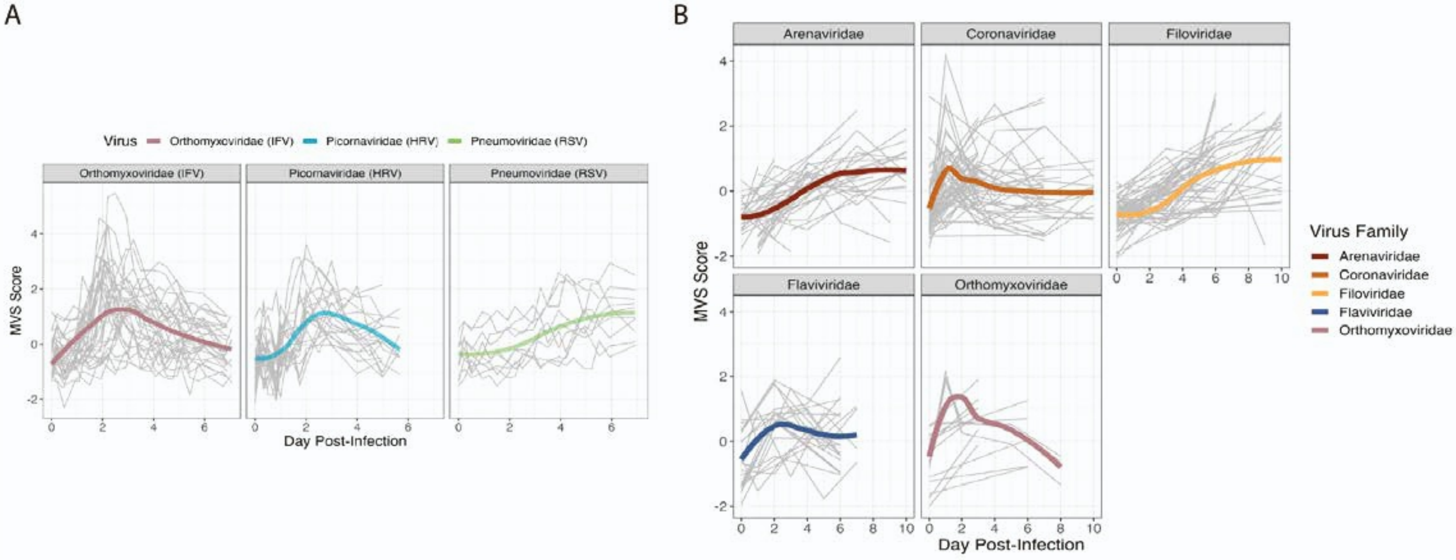

(A-B) MVS score calculated across (A) human longitudinal datasets and (B) all NHP longitudinal data.

Table S4: Time series analysis of MVS score by viral challenge in humans

Random effects:	Variance	Std. Deviation	Corr		
Participant	0.404	0.635			
Time	0.014	0.118	-0.36		
Residual	0.574	0.758			
**Fixed Effects:**	**β parameter estimate**	**Std. Error**	**t value**	**Pr(>|t|)**	
Intercept	-0.537	0.137	-3.932	1.70E-04	***
Time	0.904	0.059	15.443	< 2e-16	***
Time x Time	-0.133	0.008	-16.214	< 2e-16	***
<u>Virus</u>					
Picornaviridae (HRV)	-0.326	0.211	-1.547	0.126	
Pneumoviridae (RSV)	0.019	0.278	0.070	0.944	
<u>Virus x Time</u>					
Picornaviridae (HRV) x Time	0.145	0.112	1.288	0.198	
Pneumoviridae (RSV) x Time	-0.642	0.118	-5.465	6.45E-08	***
<u>Virus x Time x Time</u>					
Picornaviridae (HRV) x Time x Time	-0.032	0.019	-1.623	0.105	
Pneumoviridae (RSV) x Time x Time	0.134	0.016	8.234	5.43E-16	***
Number of observations:	1158				
Number of participants:	64				
AIC:	2918.891				

Mixed effects model using R package lmerTest. Comparison are to Orthomyxoviridae (IFV) challenge. Data from respiratory viral challenge of subjects with symptomatic disease. Timepoints included were from day 0 to day 7 post-virus challenge.
Signif. codes: 0 '***' 0.001 '**' 0.01 '*' 0.05

Table S5: Time series analysis of MVS score by Orthomyxoviridae challenge of humans and NHPs

Random effects:	Variance	Std. Deviation	Corr		
Subject	0.376	0.613			
Time	0.011	0.107	-0.24		
Residual	0.691	0.831			
**Fixed Effects:**	**β parameter estimate**	**Std. Error**	**t value**	**Pr(>|t|)**	
Intercept	-0.539	0.138	-3.908	2.56E-04	***
Time	0.905	0.063	14.397	< 2e-16	***
Time x Time	-0.133	0.009	-14.818	< 2e-16	***
Species					
NHP	0.153	0.289	0.530	0.597	
Species x Time					
NHP x Time	-0.003	0.250	-0.012	0.990385	
Species x Time x Time					
NHP x Time x Time	-0.010	0.045	-0.231	8.17E-01	
Number of observations:	656				
Number of participants:	48				
AIC:	1775.571				

Mixed effects model using R package lmerTest. NHP Orthomyxoviridae challenge cohorts compared to Human Orthomyxoviridae challenge cohorts. Timepoints included were from day 0 to day 7 post-virus challenge. Signif. codes: 0 '***' 0.001 '**' 0.01 '*' 0.05

SFig5. Meta-analysis of Human symptomatic challenge datasets by virus and time category

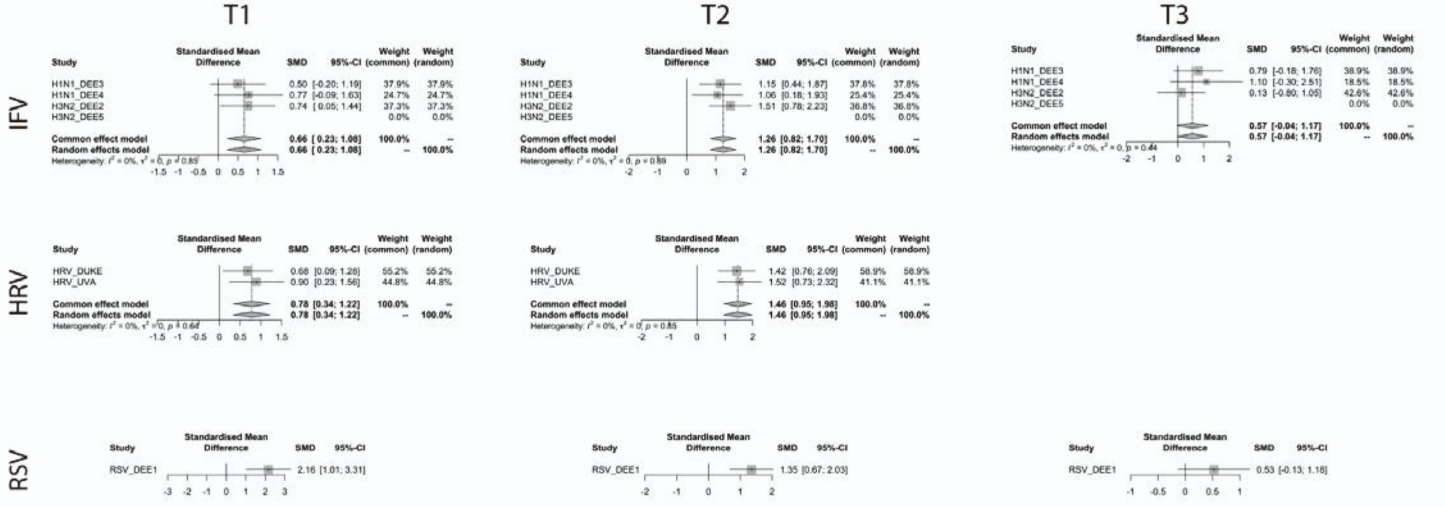

Meta-analysis of the MVS score across timepoints categories and viral infection in longitudinal human datasets.

SFig6. Meta-analysis of NHP datasets by virus and time category

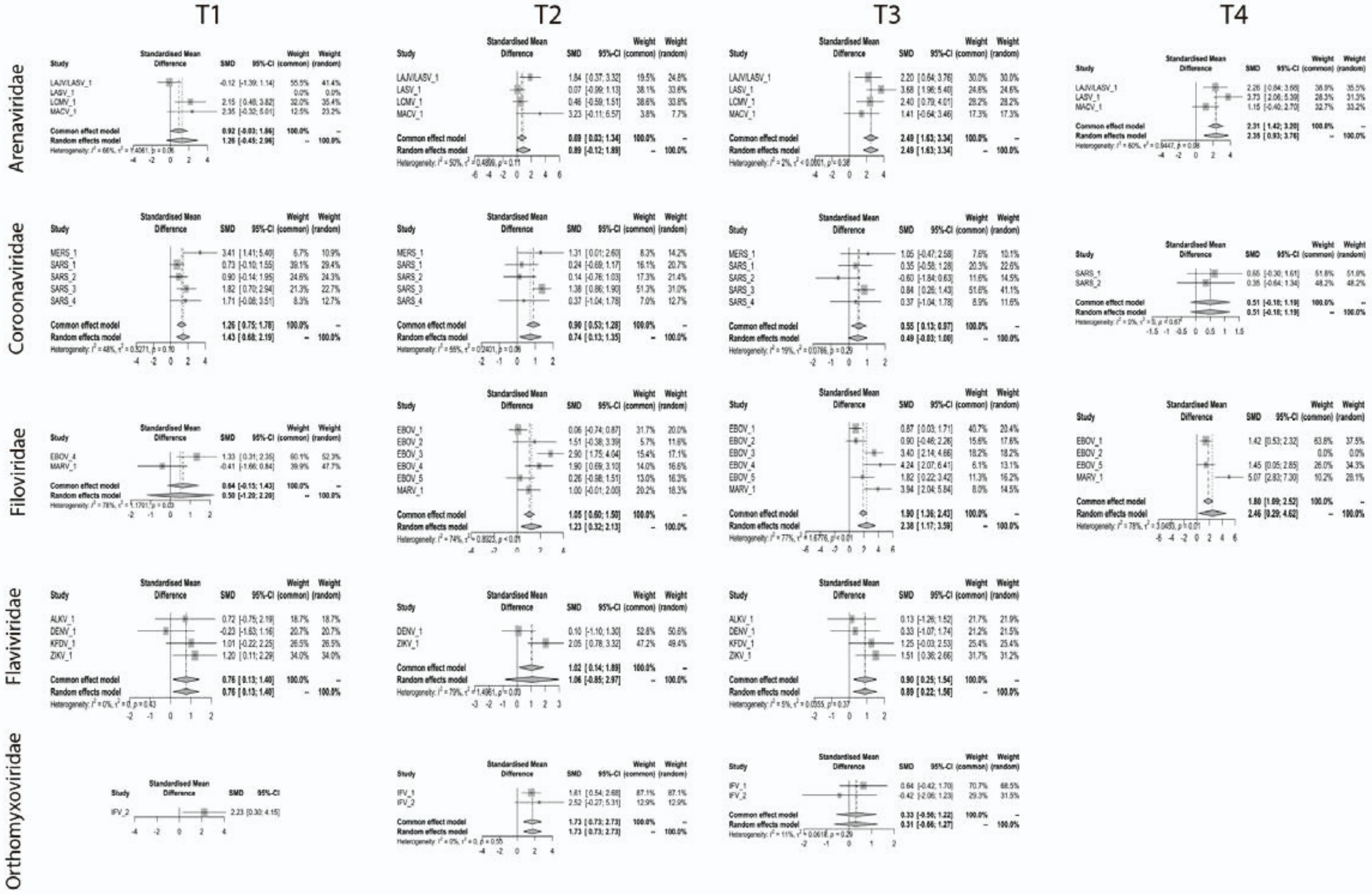

Meta-analysis of the MVS score across timepoints categories and viral infection in macaque datasets.

SFig7: BTM enrichment analysis across DEGs at peak timepoints per virus

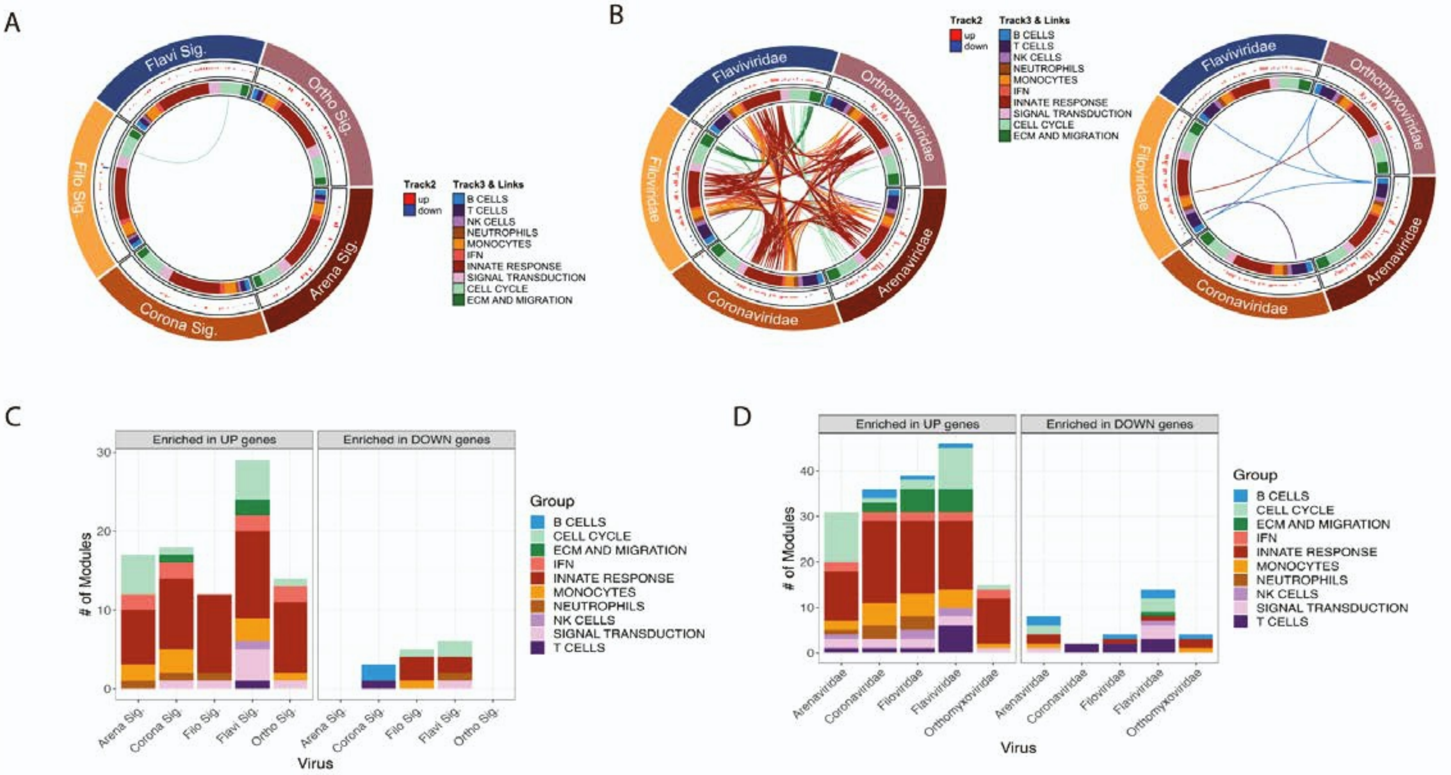

(A-C) Circos plots of BTM enrichment analysis across (A) underexpressed genes in each signature and **(B)** upregulated (left) and downregulated (right) genes at peak infection timepoints in NHP datasets. Each sector represents a viral family, each point in all the tracks represents a BTM that was significant in at least one virus (padj <0.1). Track 2 is a barplot of the geometric mean of the expression of the genes represented by the BTM and plotted where the BTM was significant (padj <0.1). Each color in Track 3 is a granular annotation for each BTM pathway. The inner track connects the same BTM across viral families if they are both (left) positively or (right) negatively enriched. **(C-D)** The count of the number of significant modules corresponding to each granular pathway by (C) overrepresented and underrepresented genes in each virus signature and (D) positive versus negative enriched genes by viral family - represented in the barplot.

SFig8. MVS and VRS in distinguishing between virus infected versus uninfected humans

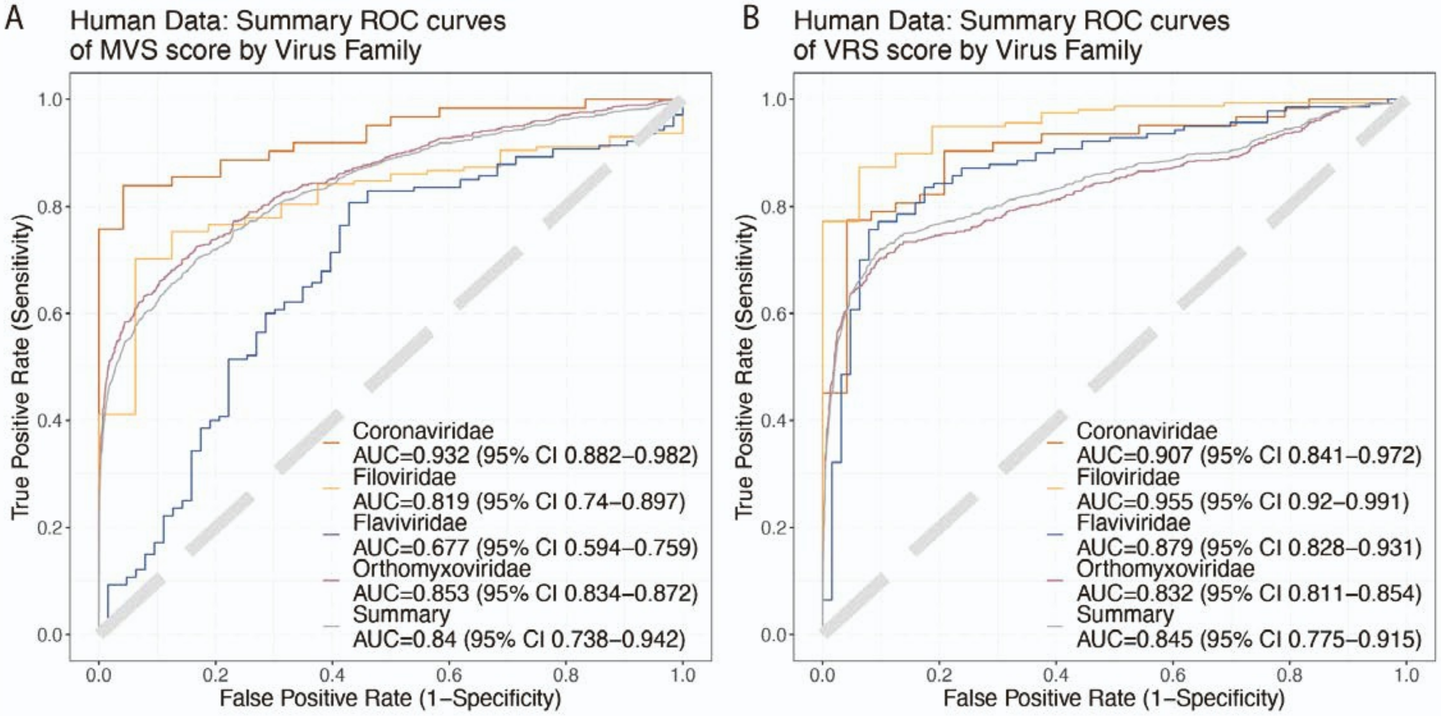

(A-B) ROC curves for distinguishing humans with viral infection from uninfected, healthy individuals split by viral family using the (A) MVS Score and (B) VRS Score.

SFig9. MVS score across human DNA and chronic viruses

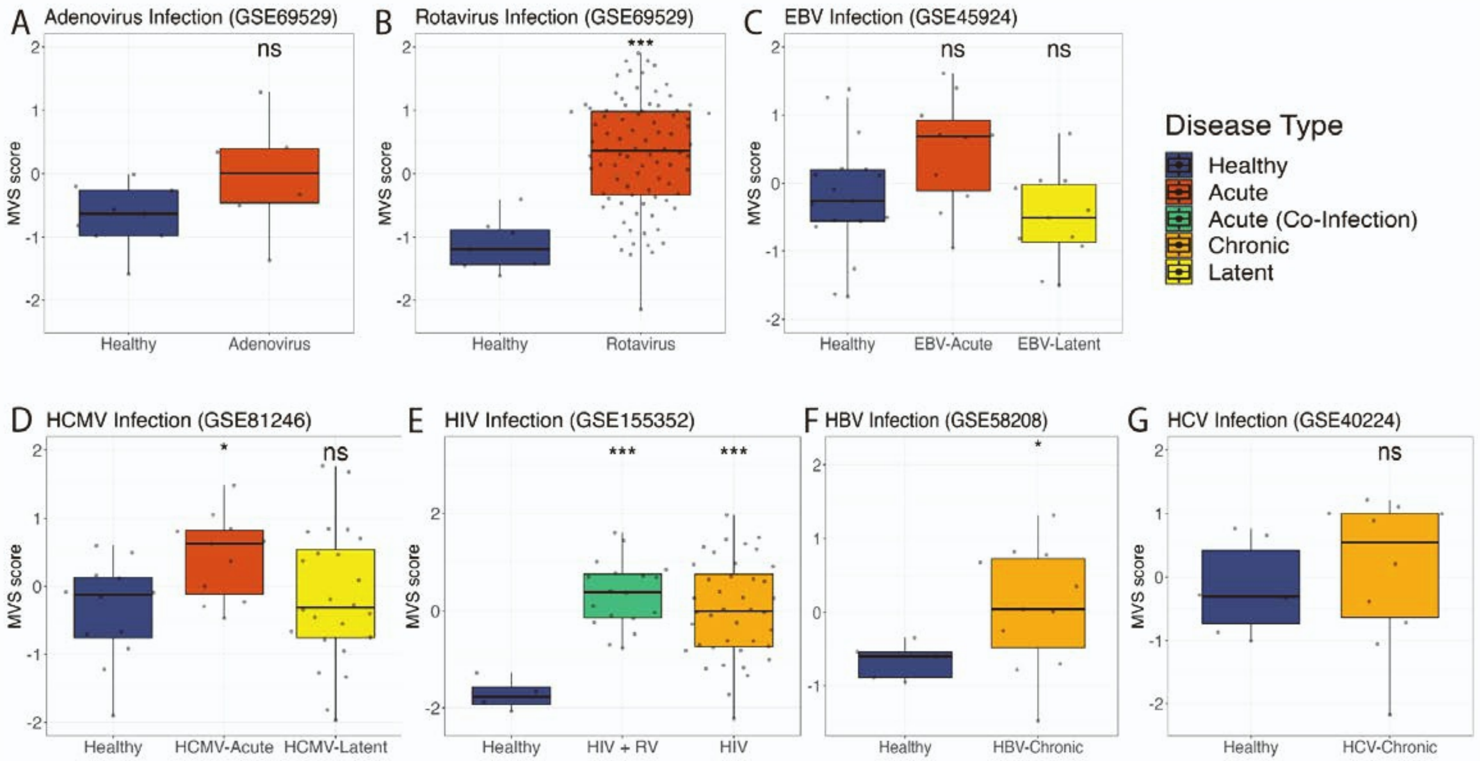

(A-G) MVS score in blood samples from healthy control subjects versus patients with **(A)** Adenovirus infection, **(B)** Rotavirus infection, **(C)** acute or latent EBV infection, **(D)** acute or latent HCMV infection, **(E)** HIV infection or HIV co-infection with a respiratory virus RV), **(F)** chronic HBV infection, and **(G)** chronic HCV infection. **(A-G)** Significance values were determined using an unpaired, one-sided Wilcoxon ranked-sum test comparing each condition to healthy samples.. Bonferroni correction for multiple hypothesis testing was applied per subfigure and significance values were assigned by asterisk. Asterisk values across figure are represented as follows: *p value < 0.05, **p value < 0.01, ***p value < 0.001, and ****p value < 0.0001. RV = respiratory virus.

SFig10: Overview of human scRNA-seq datasets

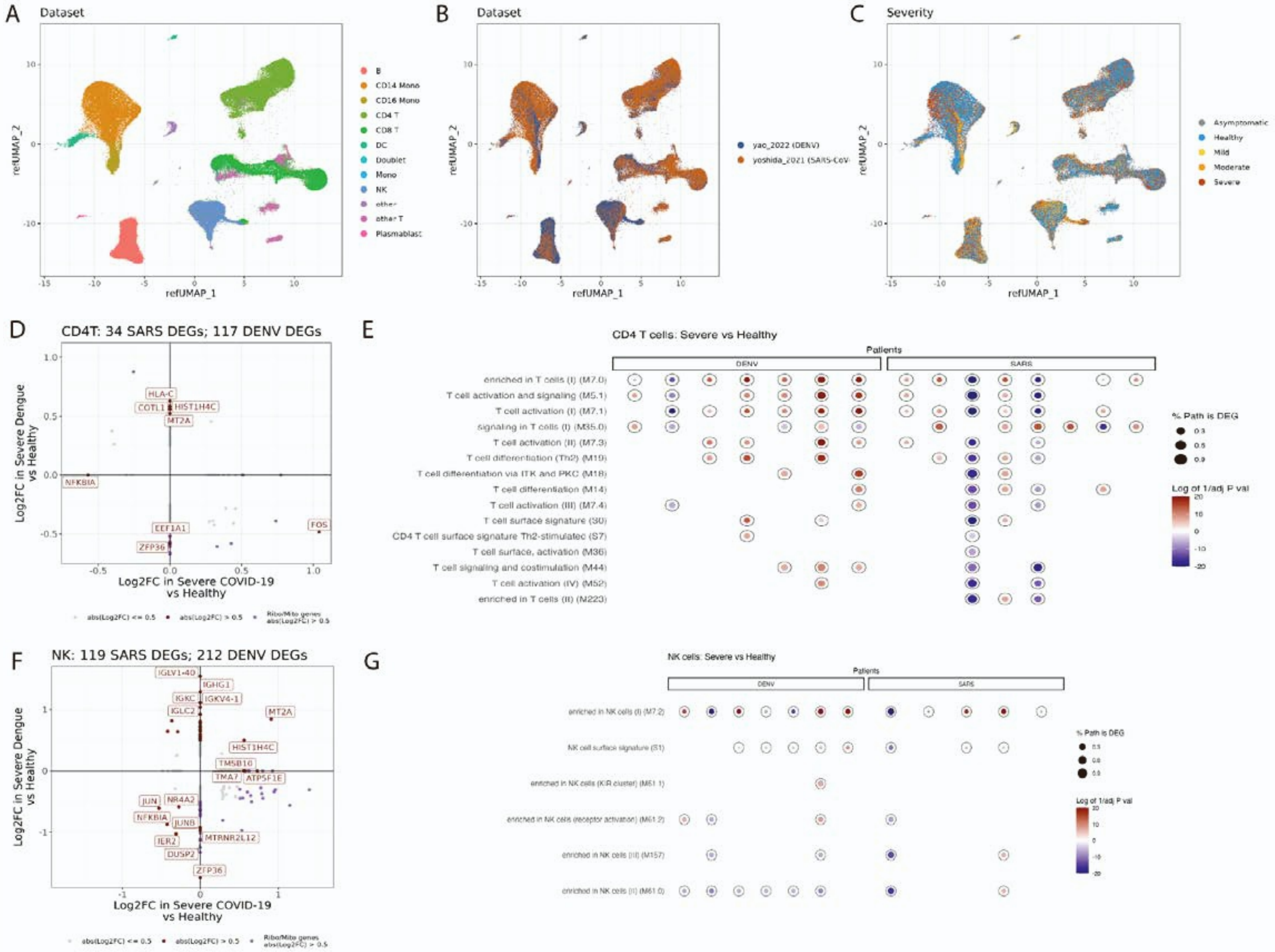

(A-C) UMAP visualization of immune cells from patients colored by **(A)** cell type, **(B)** dataset, and **(C)** patient severity. **(D and F)** Differential gene expression analysis of **(D)** CD4 T cells and **(F)** NK cells across scRNA-seq data from COVID-19 and dengue patients between patients with severe disease compared to healthy controls. **(E and G)** BTM enrichment analysis of differentially expressed genes from **(E)** CD4 T cells and **(G)** NK cells from each severe patient compared to the dataset's healthy patients.
